# Recent Progress on Enyne Metathesis: Its Application to Syntheses of Natural Products and Related Compounds

**DOI:** 10.3390/ma3032087

**Published:** 2010-03-19

**Authors:** Miwako Mori

**Affiliations:** Health Sciences University of Hokkaido, Ishikari-Tobetsu, Hokkaido 061-0293, Japan; E-Mail: mori@pharm.hokudai.ac.jp; Tel: +81 11 7876045; Fax: +81 11 7876045

**Keywords:** enyne metathesis, ring-closing enyne metathesis, dienyne metathesis, cross metathesis, ring-opening metathesis, natural product

## Abstract

Olefin metathesis using ruthenium carbene complexes is a useful method in synthetic organic chemistry. Enyne metathesis is also catalyzed by these complexes and various carbo- and heterocycles could be synthesized from the corresponding enynes. Dienyne metathesis, cross enyne metathesis and ring-opening enyne metathesis have been further developed. Various complicated compounds, such as the natural products and the related biologically active substances, could be synthesized using these metatheses reactions. Skeletal reorganization using the transition metals and metallotropic rearrangement are also discussed.

## Table of Content

Introduction
1. Ring-Closing Enyne Metathesis
2. Ring-Closing Dienyne Metathesis
3. Cross Enyne Metathesis
4. Ring-Opening Enyne Metathesis
5. Skeletal Reorganization Using Transition Metals
6. Metallotropic Rearrangement
Perspective

## Introduction

**Figure 1:**
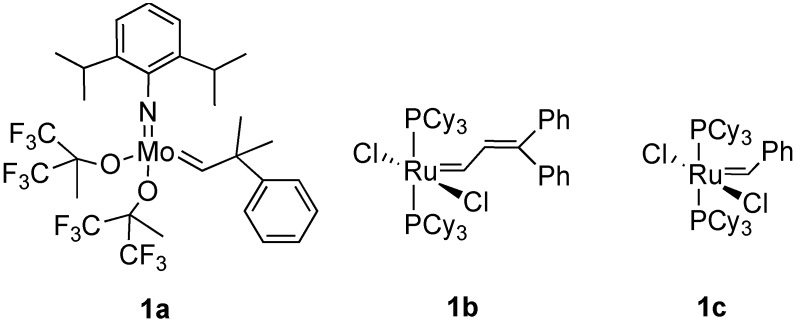
Ruthenium catalysts for alkene metathesis.

Since the discovery of molybdenum and ruthenium carbene complexes by Schrock and Grubbs in 1990 [1] and 1992 [2], synthetic organic chemistry has made rapid progress using metathesis reactions. Grubbs *et al.* found that molybdenum carbene complex **1a** was effective for olefin metathesis [3]. They then synthesized ruthenium-carbene complex **1b** for olefin metathesis [2], and synthesized carbo- and heterocyclic compounds using ring-closing olefin metathesis [4,5,6]. In 1995, Grubbs found that ruthenium-carbene complex **1c** has the same reactivity as that of **1b** [7], and it is now commercially available. Complexes **1b** and **1c** are stable and easy to handle (Figure 1). Thus, many researchers were able to use these catalysts, and various cyclic compounds were synthesized from dienes using ring-closing metathesis (RCM).

**Figure 2:**
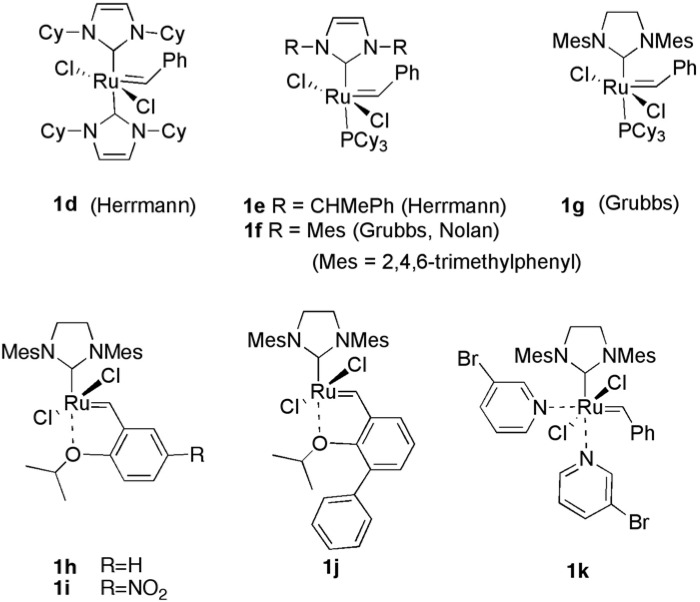
Ruthemium catalysts for alkene metathesis.

In 1999, Herrmann [8,9], Nolan [10,11] and Grubbs [12,13,14,15,16] found novel ruthenium-carbene complexes **1d-1g** having a heterocyclic carbene as a ligand. Since these catalysts, called as the second-generation ruthenium carbene complex, are very effective for olefin metathesis compared with **1b** and **1c** [14], olefin metathesis has been further progressed by use of these catalysts. Furthermore, cross-metathesis (CM) of alkene and ring-opening metathesis (ROM) have been developed using these complexes. Later, many ruthenium carbene complexes **1h-k** [17,18,19,20,21,22] having various ligands were synthesized (Figure 2).

**Scheme 1:**
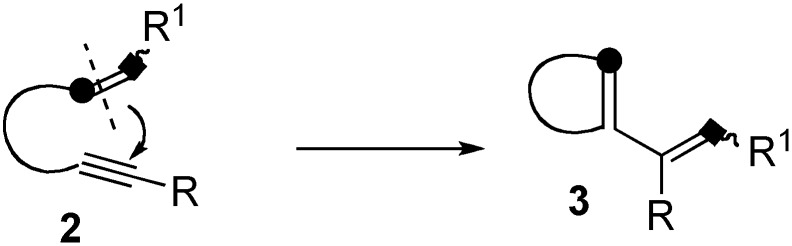
Ring-opening enyne metathesis.

Metathesis of enynes having alkene and alkyne moieties in a molecule is an extremely interesting reaction [23,24,25,26,27]. In this reaction, the double bond of enyne **2** is cleaved and a carbon-carbon bond is formed between the double and triple bonds, and the cleaved alkylidene part of the double bond migrates onto the alkyne carbon to produce a cyclic compound **3** having a 1,3-diene moiety (Scheme 1).

**Scheme 2:**
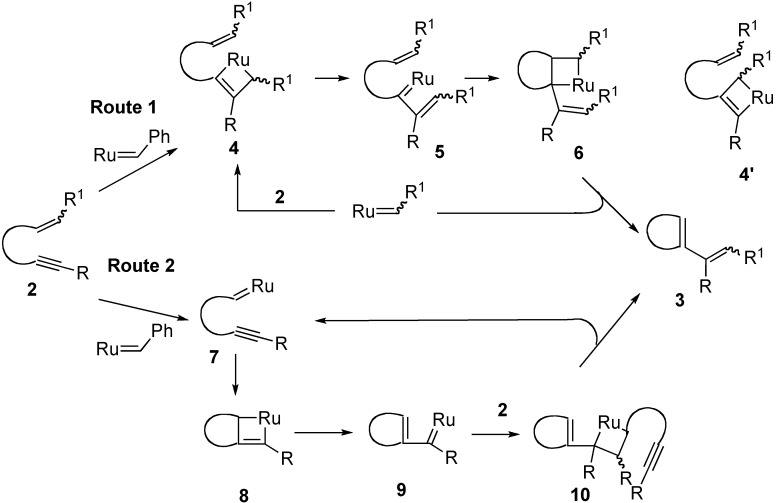
Ring-closing enyne metathesis.

The first enyne metathesis was reported by Katz [28,29,30], who used a Fischer tungsten-carbene complex. Then Mori reported a chromium-catalyzed enyne metathesis [31,32,33,34]. It was later found that the ruthenium-carbene complex **1b** or **1c** was very effective for enyne metathesis [35,36]. The reaction would proceed *via* a [2+2] cycloaddition of a ruthenium-carbene complex with an alkyne part to produce ruthenacyclobutene **4**, and ring-opening of this affords a ruthenium carbene complex **5**, which reacts with an alkene part to produce ruthenacyclobutane **6**, and ring-opening of this gives a cyclized compound **3** and a ruthenium-carbene complex is regenerated (Scheme 2, Route 1). In some cases, metathesis products *via* complexes **4’** were obtained. The other mechanism also considered involves at first reaction of the alkene part of enyne **2** with ruthenium carbene complex to afford a new ruthenium carbene complex **7**. The latter species reacts with the alkyne part to produce ruthenacyclobutene **8** and its subsequent ring-opening gives ruthenium carbene **9**, that undergoes intermolecular [2+2] cycloaddition with the alkene part of enyne **2** to produce ruthenacyclobutane **10**. Ring-opening of this gives cyclic compound **3** and ruthenium carbene complex **7** is regenerated (Scheme 2, Route 2).

Later, the detailed study on the reaction mechanism was shown by Lippstreu and Straub, who described that the reaction would proceeds *via* Route 2, and ruthenacyclobutene **4**, generated from an alkyne part of enyne **2** and ruthenium carbene complex, do not exist as local minimum in the catalytic cycle [37].

Using ruthenium carbene complexes **1b** and **1c**, various carbo- and heterocycles could be synthesized from the corresponding enynes [35,36]. Furthermore, dienyne metathesis, cross enyne metathesis and ring-opening enyne metathesis have been developed. As the results, the novel route for the synthesis of various complicated compounds, such as the natural products and the related biologically active substances, were pioneered. Skeletal reorganization using the transition metals and metallotropic rearrangement will be discussed.

## 1. Ring-Closing Enyne Metathesis (RCM)

**Scheme 3:**
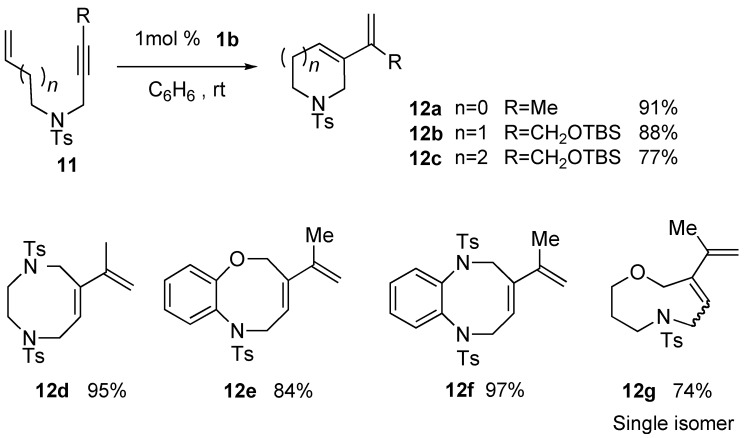
Synthesis of heterocycles using ruthenium catalyst.

Mori reported the synthesis of heterocycles having a diene moiety using enyne metathesis [35,36]. Enynes **11** were treated with 1 mol % of Grubbs catalyst **1b** at room temperature to afford heterocycles **12** in high yields. Using this procedure, five- to nine-membered heterocycles could be synthesized (Scheme 3).

**Scheme 4:**
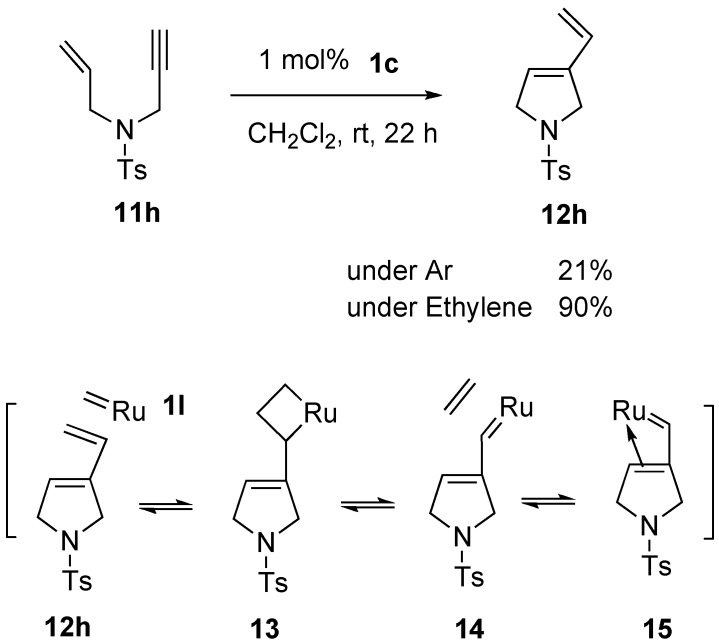
Metathesis of enyne having terminal alkyne under ethylene gas.

In this reaction, enynes **11** (R = H), having a terminal alkyne, did not give a satisfactory result [38]. For example, RCM of enyne **11h** afforded cyclic compound **12h** in only 21% yield. It is reasoned that an alkene part in product **12h** further reacts with ruthenium carbene methylidene complex **1l** to afford ruthenium carbene **14**, which would be coordinated by the alkene part to produce **15**. Thus, the catalytic activity would decrease (Scheme 4). In fact, when the reaction of **11h** using **1c** was carried out under ethylene gas, the catalytic activity was much larger to afford **12h** in 90% yield, even with the use of 1 mol % of the ruthenium catalyst **1c**. The higher reactivity observed in enyne metathesis in the presence of ethylene gas has often been advantageous in applications to natural product synthesis.

**Scheme 5:**
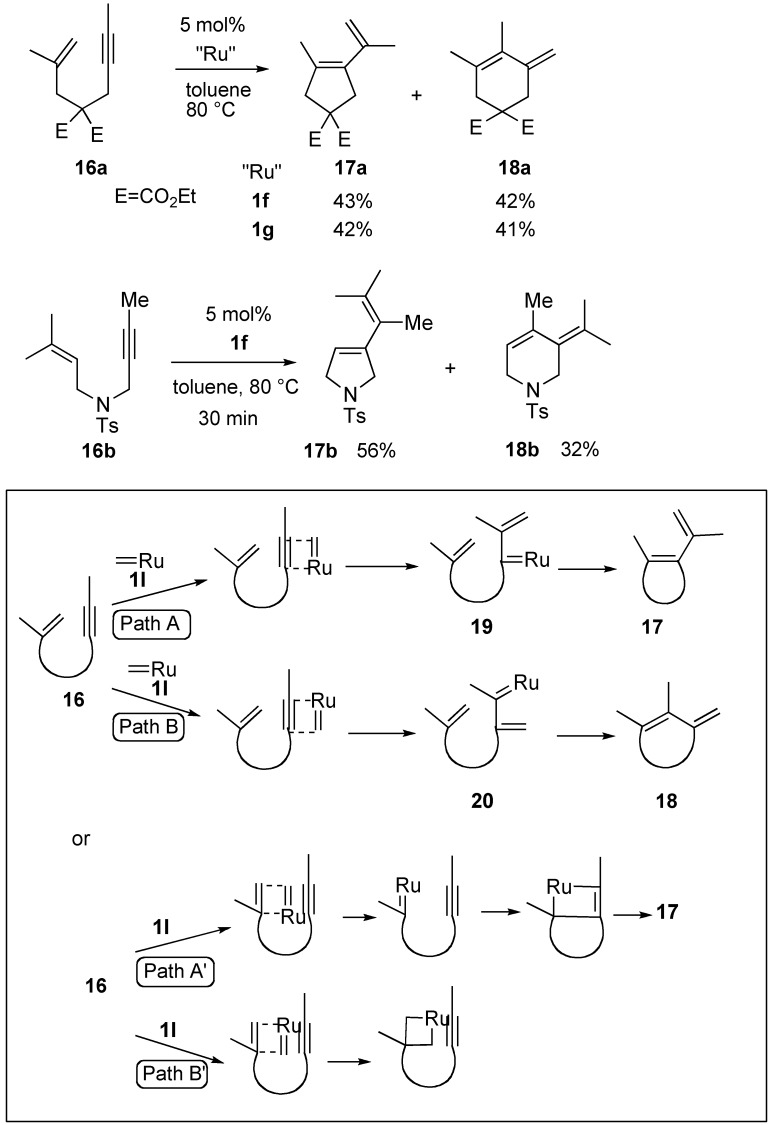
Enyne metathesis using second generation ruthenium catalysts.

When the second-generation ruthenium carbene complexes were used for enyne metathesis, unprecedented results were shown [39,40]. The reaction of enyne **16a** or **16b** was carried out using 5 mol % of **1f** to afford expected metathesis product **17a** or **17b** along with six-membered compound **18a** or **18b**. Use of other second-generation ruthenium carbene complex **1g** to the reaction of **16a** gave a similar result. Presumably, when ruthenium carbene methylidene complex **1l** reacts with the alkyne moiety in **16**, two regiochemically different pathways are possible (Scheme 5, path A and B). Each carbene complex **19** or **20** gives a different product **17** or **18**, respectively. On the other hand, if **1l** reacts with an alkene moiety (path A’), compound **17** is formed. In this case, the path B’ is a non-productive process. Thus, compound **18** should be formed *via* path B. However, it is not clear why two products **17** and **18** were formed when the second-generation ruthenium carbene complexes were used.

**Scheme 6:**
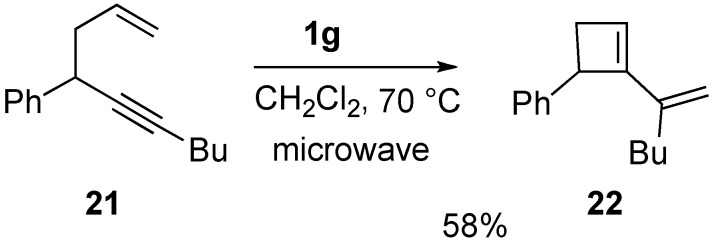
1,5-Enyne metathesis.

Recently, it was reported that cyclobutene derivative **22** could be synthesized from enyne **21** using ruthenium carbene complex **1g** under microwave irradiation conditions with 58% yield (Scheme 6) [41].

**Scheme 7:**
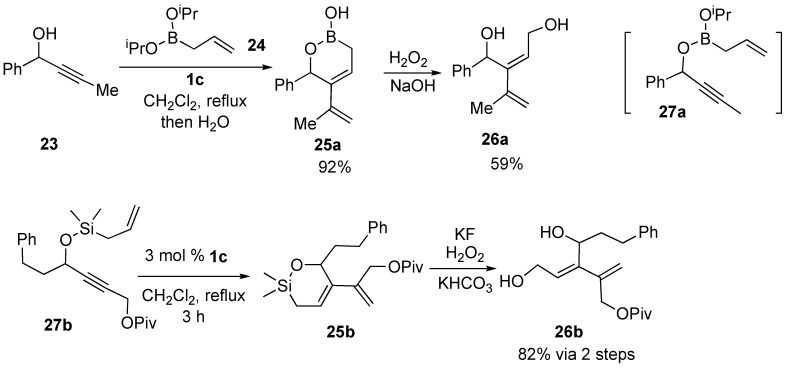
Synthesis of functionalized diene.

Two synthetic methods of highly functionalized conjugated diene **26** using ring-closing enyne metathesis were reported (Scheme 7). The reaction of propargyl alcohol **23** and allylboronate **24** in the presence of **1c** gave cyclic boronic ester **25a**. In this reaction, enyne **27a** should be formed as an intermediate. Treatment of cyclic boronic ester **25a** with H_2_O_2_ in aqueous NaOH gave diol **26a** [42,43]. Silicon-tethered ring closing enyne metathesis of **27b** by ruthenium carbene catalyst **1c** gave **25b**, which was subjected to Tamao oxidation to afford diol **26b** [44,45].

**Scheme 8:**
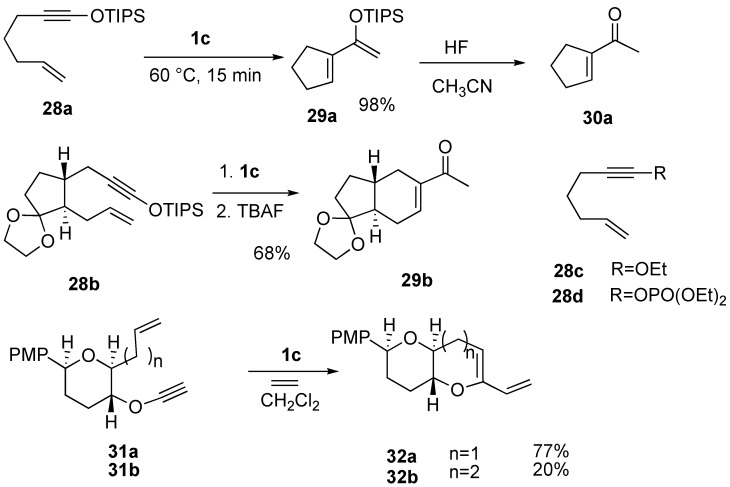
Metathesis of ene-alkynyl ether.

Enyne **28a** having a silyl ynol ether on the alkyne gave cyclic compound **29a** having a silyl enol ether moiety, which was converted into 1-acetylcyclopentene **30a** by desilylation. In a similar manner, enyne **28b** afforded bicyclic methyl ketone **29b** in 68% yield after deprotection of the silyl group [46]. However, ynoate **28c** and yne-phosphonate **28d** did not give cyclized compounds. Ene-alkynyl ether **31a** or **31b** afforded cyclic enol ethers **32a** or **32b** in good to moderate yield (Scheme 8) [47].

**Scheme 9:**
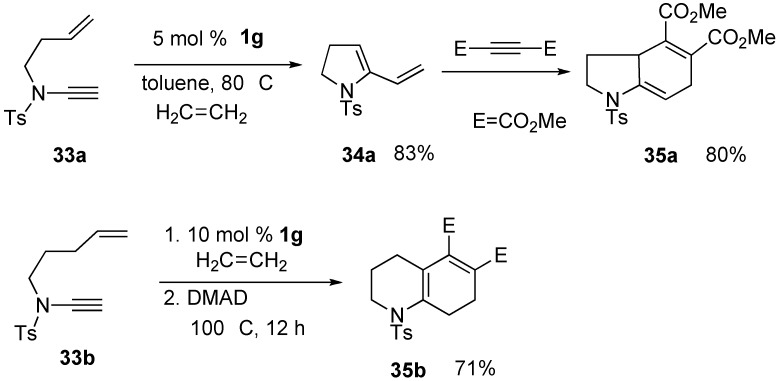
Ring-closing metathesis of ene-alkynyl amide.

RCM of ene-alkynyl amide **33a** using **1g** gave pyrrolidine derivative **34a**, which afforded indole derivative **35a** by Diels–Alder reaction [48,49]. In a similar manner, one-pot RCM of ene-alkynyl amide **33b**, a one carbon-elongated homolog, followed by Dield–Alder reaction, gave quinoline derivative **35b** in a high yield (Scheme 9) [48,49,50,51].

**Scheme 10:**
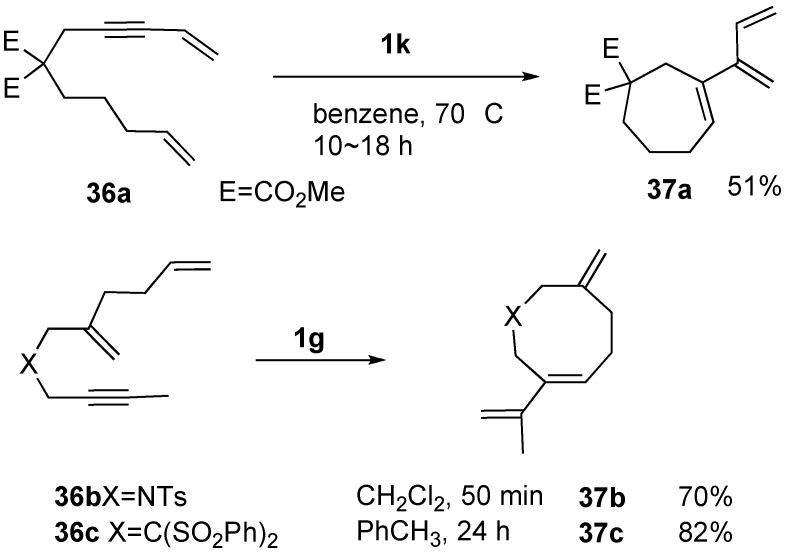
Reaction of enyne containing diene-yne.

Metathesis of enyne **36a** containing conjugated ene-yne using ruthenium catalyst **1k** gave cycloheptene derivative **37a** having a triene unit. This reaction did not proceed by first or second-generation ruthenium catalyst **1c** or **1g** (Scheme 10) [52]. Enynes **36b** and **36c** having internal alkyne and diene afforded eight-membered ring compounds **37b** and **37c** in high yields [53].

**Scheme 11:**
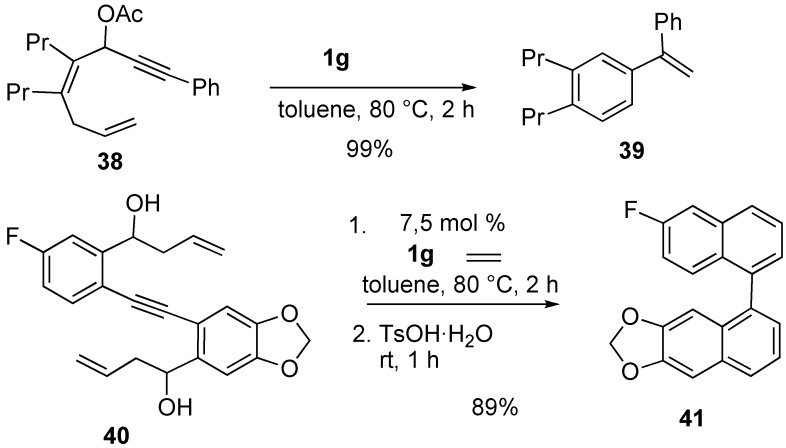
Synthesis of carbocyclic aromatic ring-closing metathesis.

The synthesis of substituted styrene **39** was achieved by ring-closing enyne metathesis (Scheme 11). As an application of this method, 1,1’-binaphthyl derivative **41** was prepared [54,55].

**Scheme 12:**
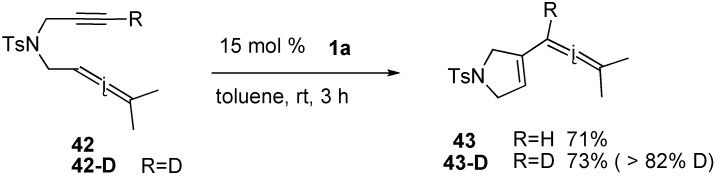
Ring-Closing Enyne Metathesis (RCM) of allene-yne

Metathesis of allene-yne **42** using molybdenum catalyst **1a** gave cyclic compound **43** having an allene moiety. RCM of **42**-**D** gave **43**-**D**, which indicates that the vinylallene skeleton was constructed by a metathesis-type reaction between the alkyne moiety and the proximal carbon-carbon double bond of the allene moiety (Scheme 12) [56].

### Syntheses of Natural Products and Related Compounds Using Ring-Closing Enyne Metathesis (RCM)

**Scheme 13:**
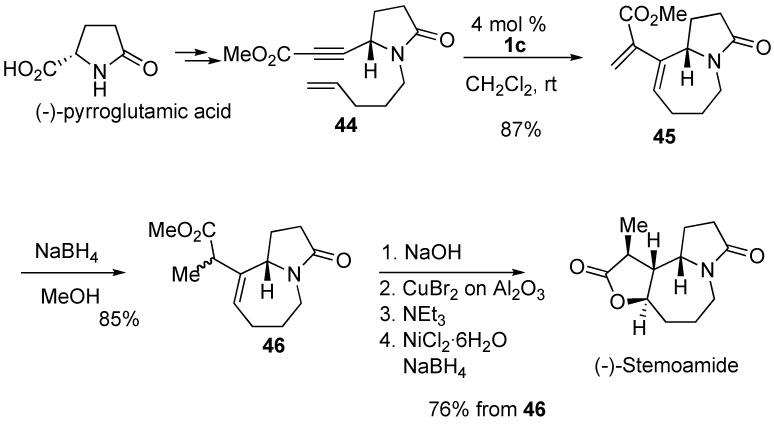
Total synthesis of (-)-stemoamide.

The first example of the total synthesis of a natural product using ring-closing enyne metathesis is the synthesis of (-)-stemoamide [57,58]. (-)-Pyroglutamic acid was converted into enyne **44** having an ester group on the alkyne, and RCM of enyne **44** was carried out in the presence of 4 mol % of ruthenium-carbene complex **1c** to afford bicyclic compound **45** in 87% yield. Conversion of **45** into **46** smoothly proceeded. From this compound **46**, (-)-stemoamide could be synthesized (Scheme 13).

**Scheme 14:**
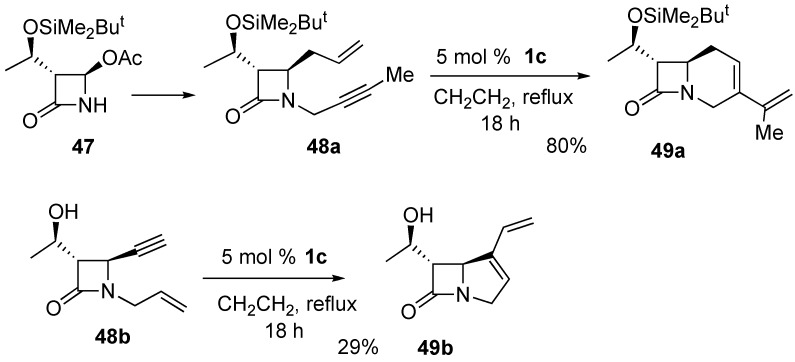
Construction of carbacephem and carbepenem skeleton.

**Scheme 15:**
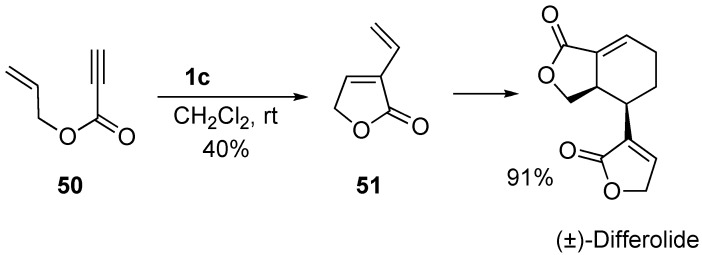
Synthesis of (±)-differolide.

Carbacephem **49a** and carbapenem **49b** were synthesized from enynes **48a** and **48b**, which were prepared from 4-acetoxy-azetidinone **47**. The yield of the latter compound **49b** is lower compared with that of **49a** because of the highly strained fused 4,5-membered ring system (Scheme 14) [59,60].

(±)-Differolide could be easily synthesized by enyne metathesis [61]. Enyne **50** was reacted with **1c** to give lactone **51**, which was spontaneously dimerized to afford (±)-differolide (Scheme 15).

**Scheme 16:**
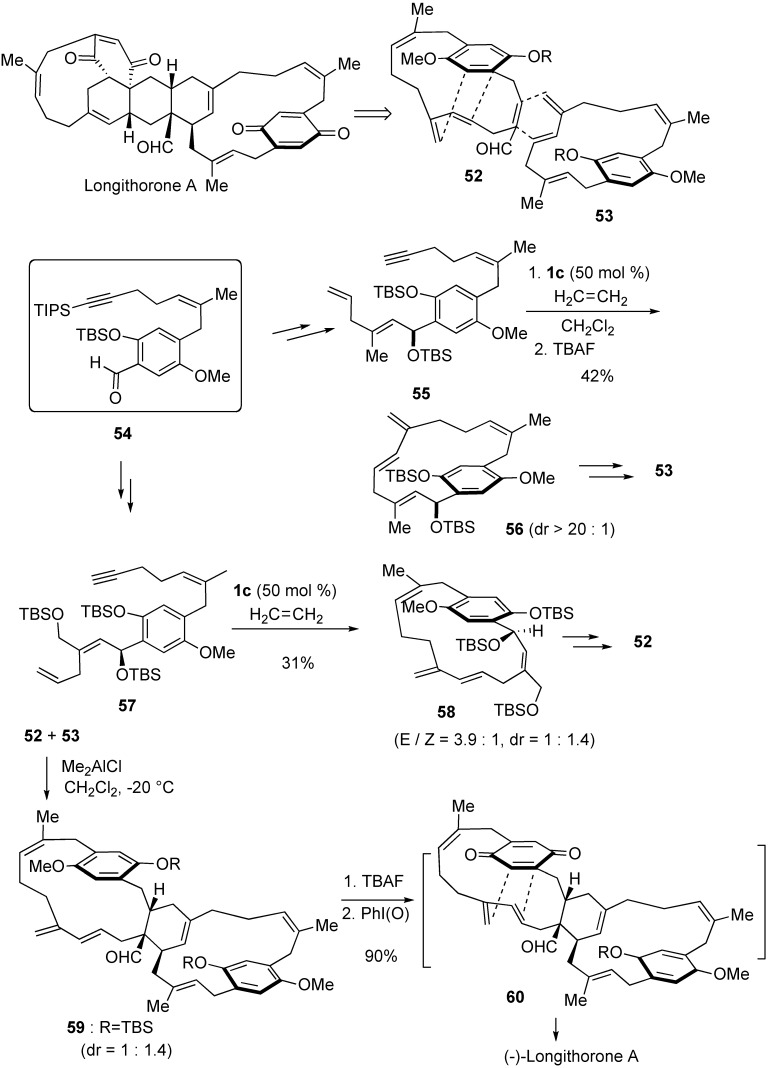
Total synthesis of (-)-longithorone A.

An enantioselective biomimetic synthesis of longithorone A was accomplished on the basis of the proposed biosynthesis [62]. Two [12]-paracyclophanes **52** and **53** were synthesized from common intermediate **54** by applying enyne metathesis macrocyclization in 42% and 31% yields, respectively. Intermolecular Diels-Alder reaction of **52** and **53** provided **59**. Deprotection followed by oxidation gave **60**, which spontaneously gave longithorone A *via* transannular Diels-Alder reaction (Scheme 16).

**Scheme 17:**
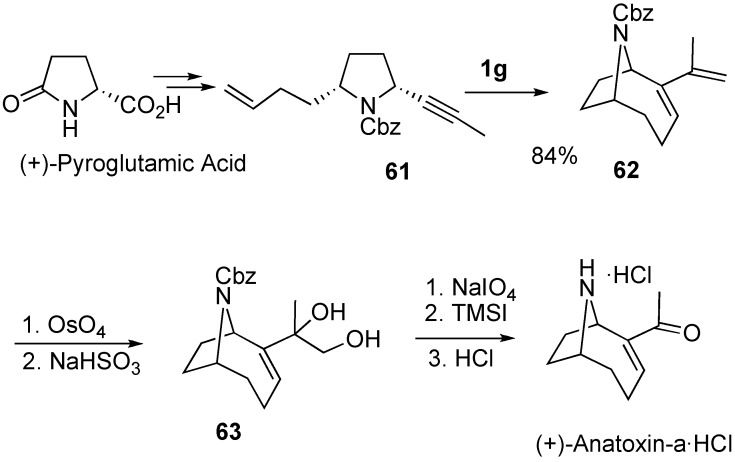
Total synthesis of anatoxin-A.

The total synthesis of (+)-anatoxin-a was achieved by Martin [63,64] and Mori [65,66] by the same strategy. The key step is the construction of an azabicyclo[4.2.1]nonene ring system. For that purpose, the 2,5-cis-disubstituted pyrrolidine derivative **61** having cis-substituents was synthesized from (+)-pyroglutamic acid. Enyne metathesis of **61** was carried out using **1g** to form this ring system. From this azabicyclic compound **62**, anatoxin-a could be synthesized (Scheme 17).

**Scheme 18:**
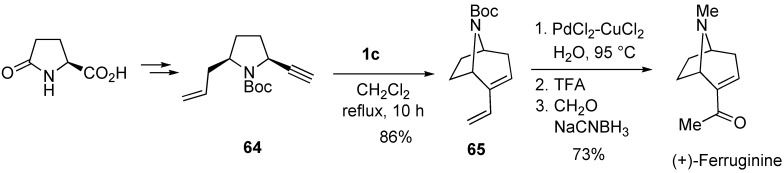
Synthesis of (+)-ferruginine.

By a similar procedure, (+)-ferruginine was synthesized from (-)-pyroglutamic acid [67]. Construction of the azabicyclo[3.2.1] octane ring system was carried out using enyne metathesis of **64**. Wacker oxidation to the resultant diene **65** afforded methyl ketone, and then deprotection followed by methylation gave (+)-ferruginine (Scheme 18).

**Scheme 19:**
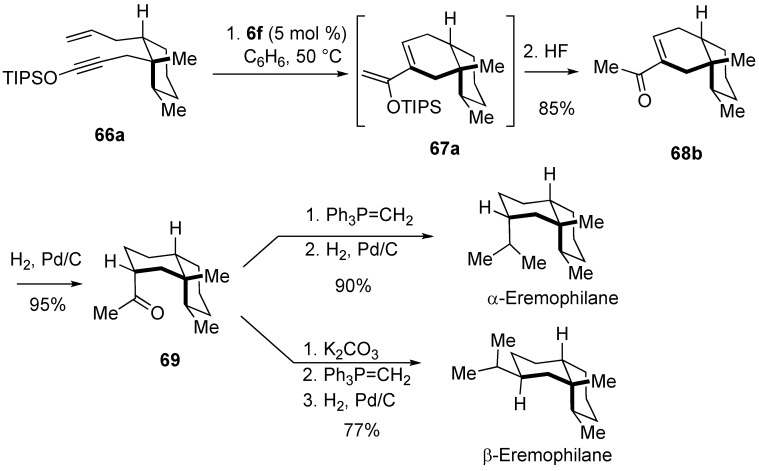
Total synthesis of eremophilane.

Kozmin developed a highly efficient synthesis of cyclic compound bearing the methyl ketone functionality from enyne having a silyloxy group on the alkyne (Scheme 8) [46]. As an application of this method, they succeeded in the synthesis of eremophilane [68]. RCM of enyne **66a** having a silyloxy group on the alkyne followed by treatment with HF gave cycloalkene **68b** having the methyl ketone functionality. Hydrogenation of the double bond in compound **68b** gave **69**. From this compound **69**, α- and β-eremophilane could be synthesized (Scheme 19).

**Scheme 20:**
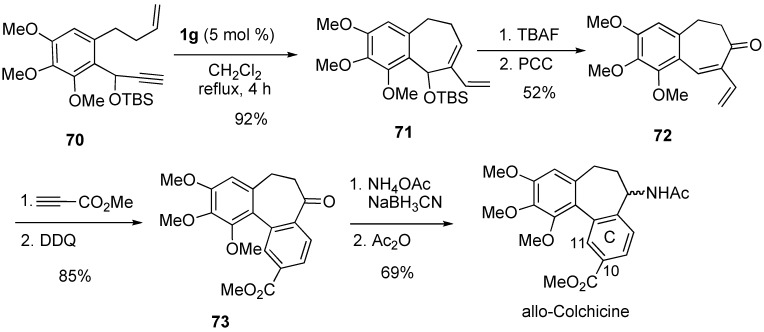
Synthesis of allo-colchicine.

New allocolchinoids functionalized at C10 or C11 in the C ring were synthesized using the RCM of enyne **70** for the construction of the seven-membered ring. The reaction proceeded smoothly to give **71** using **1g** in 92% yield. Deprotection followed by PCC oxidation gave **72**, which was subjected to a Diels-Alder-aromatization sequence to form **73**. Amination of **73** followed by acetylation gave allocholchicines (Scheme 20) [69].

**Scheme 21:**
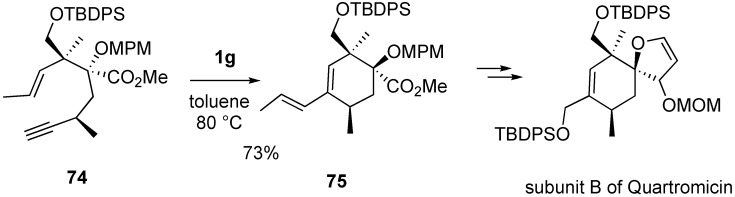
Synthesis of subunit of quartromicin.

The *agalacto*-spirolactone B subunit of quartromicins has been synthesized using the Claisen-Ireland/ RCM of enyne approach by Haudrechy *et al*. [70]. Enyne **74** was treated with **1g** in toluene at 80 °C to afford **75** in 73% yield. From this compound **75**, subunit B of quartromicin was synthesized (Scheme 21).

**Scheme 22:**
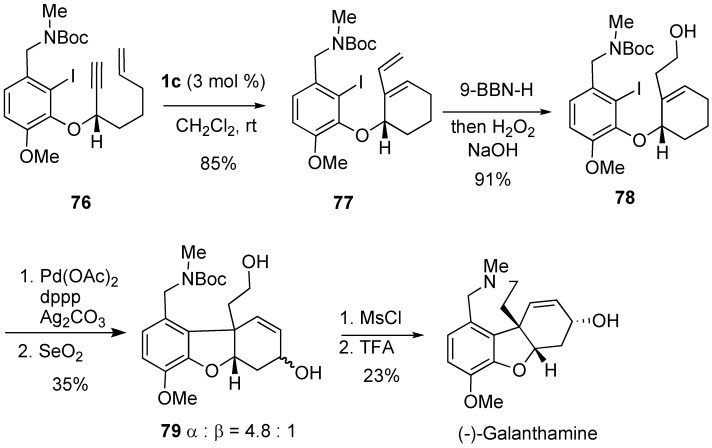
Total synthesis of galanthamine.

An enantioselective synthesis of (-)-galanthamine was realized in 11 steps starting from isovanillin (Scheme 22). The enyne **76** (92% ee) underwent an efficient RCM reaction in the presence of 3 mol % of **1c** to give diene **77** in 85% yield. Hydroboration of the terminal alkene of **77** followed by oxidation gave homoallylic alcohol **78** in excellent yield, and palladium-catalyzed cyclization followed by SeO_2_ oxidation gave **79**. Mesylation of **79** followed by deprotection afforded (-)-galanthamine [71].

**Scheme 23:**
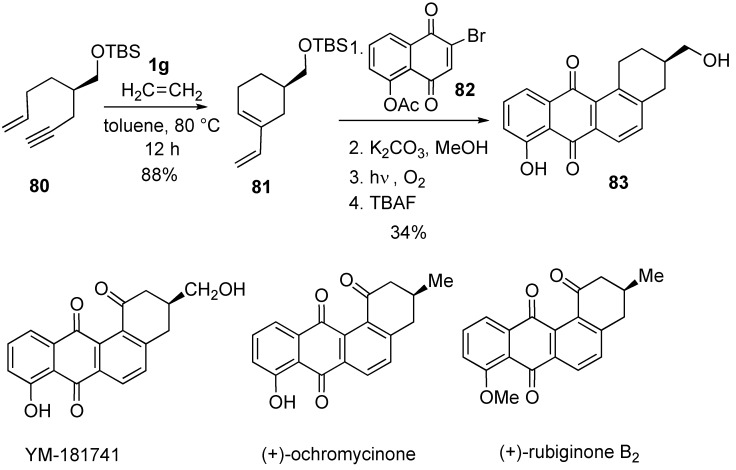
Synthesis of benz[a]anthraquinone skeleton.

For the synthesis of angucyclinone-type natural products, a concise and highly enantioselective route was developed (Scheme 23). Chiral vinylcyclohexene derivative **81** was synthesized using RCM of enyne **80** under an atmosphere of ethylene in high yield. Intermolecular Diels-Alder reaction of **81** and **82** followed by aromatization gave compound **83** having benz[a]anthraquinone skeleton. Utilization of this strategy, total synthesis of YM-181741, (+)-ochromycinone, (+)-rubiginone B have been accomplished [72].

**Scheme 24:**
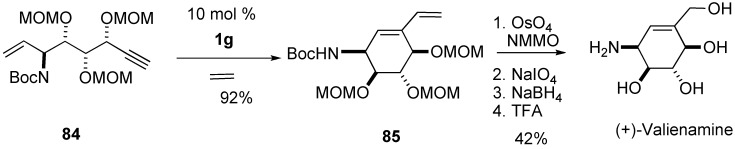
Synthesis of (+)-valienamine.

Stereoselective total synthesis of (+)-valienamine was reported utilizing ring-closing enyne metathesis (Scheme 24) [73].

An interesting acceleration effect of an allylic hydroxy group on ring-closing enyne metathesis has been demonstrated. Ring-closing enyne metathesis of terminal alkynes possessing an allylic hydroxy group proceeded smoothly. The synthesis of (+)-isofagomine with the aid of this efficient reaction has been demonstrated [74].

**Scheme 25:**
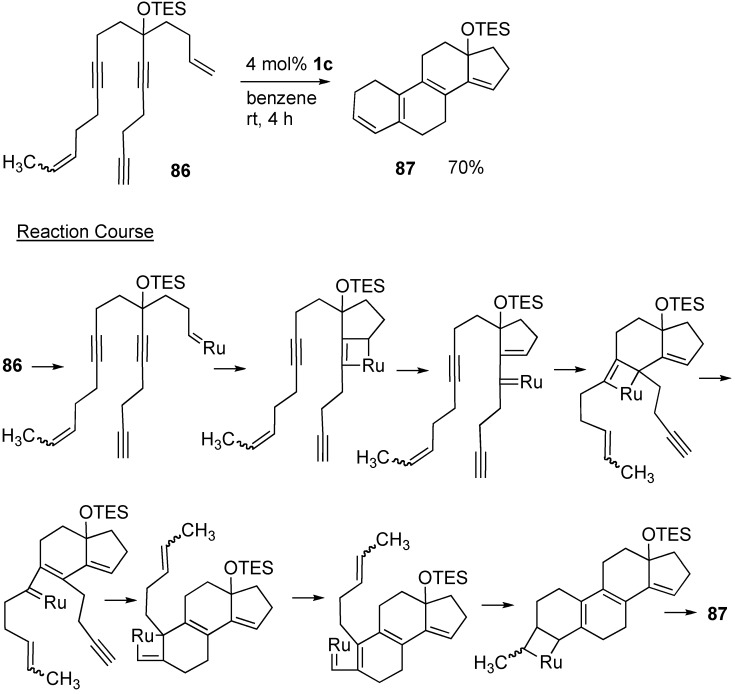
Construction of a steroidal skeleton.

Grubbs demonstrated the synthesis of various fused polycyclic compounds using tandem metathesis reaction. A steroidal skeleton could be constructed using tandem enyne metathesis. Treatment of polyenyne **86** having double and triple bonds at the appropriate positions in the carbon chain gave compound **87** in high yield in one step, although many processes were contained in this reaction as shown in Scheme 25 [75].

**Scheme 26:**
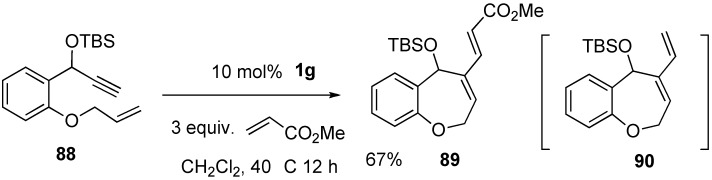
One-pot reaction of RCM-CM.

Tandem ring-closing enyne metathesis followed by cross olefin metathesis is interesting and useful because the reaction process is shortened and the yield is raised compared with that of the stepwise reaction. One-pot RCM–CM reaction was realized by Royer *et al.* (Scheme 26). The RCM of **88** in the presence of an excess amount of methyl acrylate using ruthenium carbene complex **1g** gave cyclic compound **89** in good yield. The reaction would proceed via the formation of **90** and subsequent CM with methyl acrylate to produce **89** [76].

**Scheme 27:**
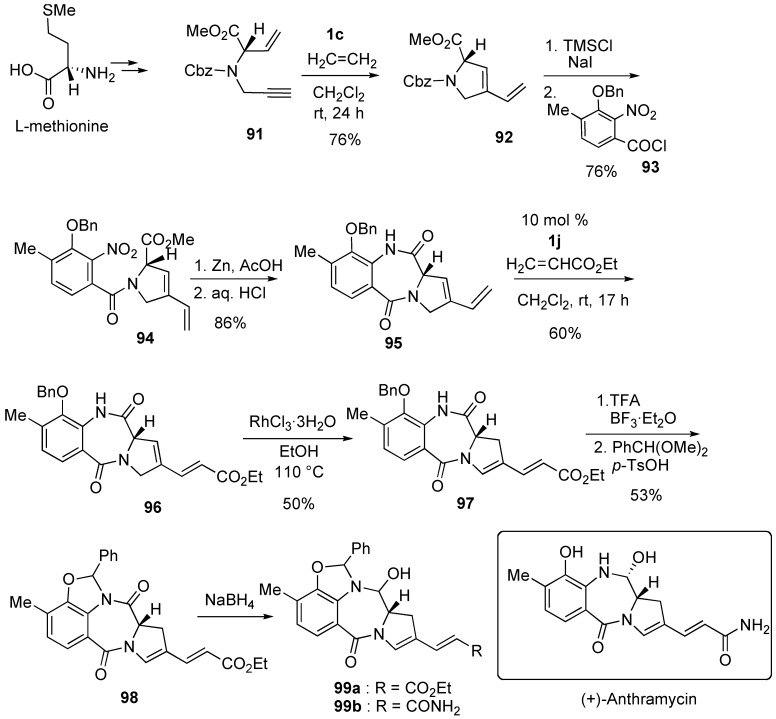
Synthesis of anthramycin derivative.

Synthesis of anthramycine derivative **99a** was achieved using RCM and CM (Scheme 27) [77]. L-Methionine was converted into enyne **91**, and RCM of **91** using **1c** gave pyrrolidine derivative **92**. Deprotection followed by condensation with commercially available acid chloride **93** gave **94**. Reductive cyclization of **94** using Zn-AcOH followed by treatment with dilute HCl gave pyrrolo-1,4-benzodiazepinone **95**. To convert the vinyl group into an α,β-unsaturated ester group, olefin cross metathesis with ethyl acrylate was carried out using catalyst **1j** [20,21]. The reaction proceeded smoothly to give compound **96** in 60% yield. Isomerization of the double bond in the pyrrolidine ring using RhCl_3_·H_2_O afforded desired compound **97**, the amide group of which was converted into aminal **98**. Conversion of **98** into ester **99a** was achieved. Stille has already reported the conversion of **99b** into (+)-anthramycin.

**Scheme 28:**
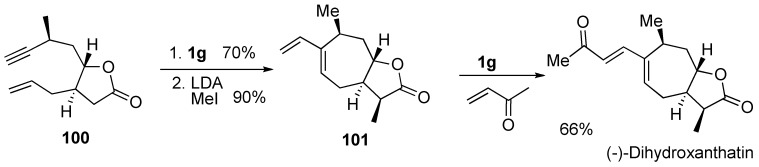
Synthesis of (-)-dihydroxanthatin.

Morken and Evans succeeded in the synthesis of (-)-dihydroxanthatin using RCM and CM [78]. Enyne metathesis of **100** using **1g** followed by methylation gave bicyclic compound **101**. Cross metathesis of **101** and methyl vinyl ketone in the presence of **1g** afforded (-)-dihydroxanthatin (Scheme 28).

**Scheme 29:**
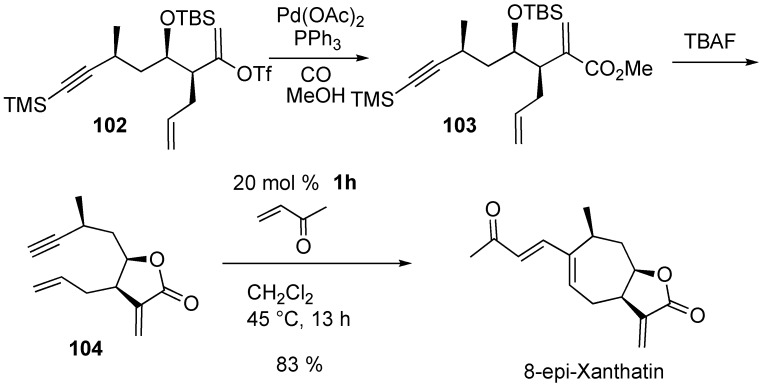
Synthesis of 8-epi-xanthatin.

Martin succeeded in the first total synthesis of the novel sesquiterpene 8-epi-xanthatin [79]. Palladium-catalyzed carbonylation of **102** followed by desilylation gave lactone **104**. The total synthesis of 8-epi-xanthatin was directly achieved by tandem RCM-CM of lactone **104** using **1h** in the presence of an excess amount of methyl vinyl ketone in one step (Scheme 29).

**Scheme 30:**
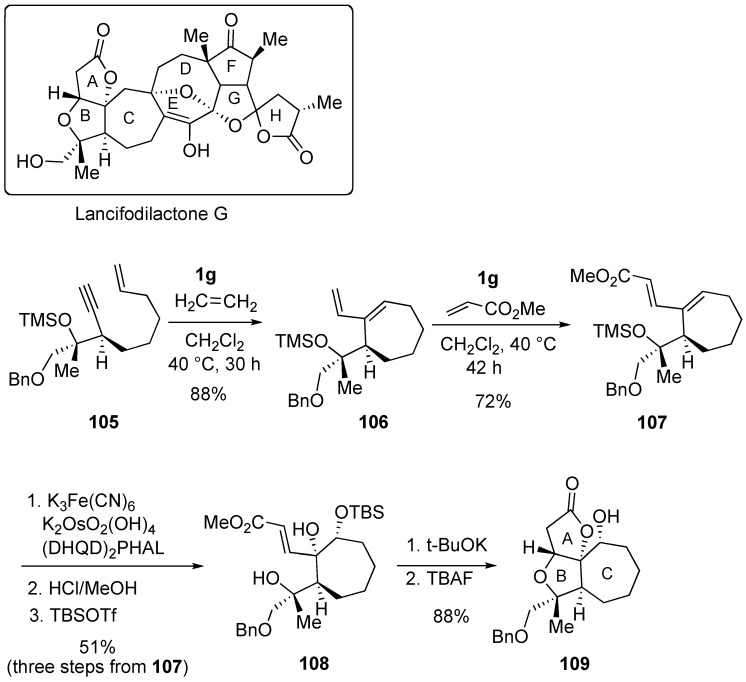
Construction of ABC ring of lancifodilactone G.

Lancifodilactone G has eight rings with complicated cyclic connectivity. Paquette synthesized ABC segment **109** of lancifodilactones G using ring-closing enyne metathesis and then cross metathesis as the key steps (Scheme 30) [80]. Ring-closing enyne metathesis of **105** using **1g** in CH_2_Cl_2_ gave cycloheptene derivative **106**, which was subjected to cross metathesis with methyl acrylate to give α,β-unsaturated ester **107**. From this compound **107**, target compound **109** was obtained.

## 2. Ring-Closing Dienyne Metathesis

**Scheme 31:**
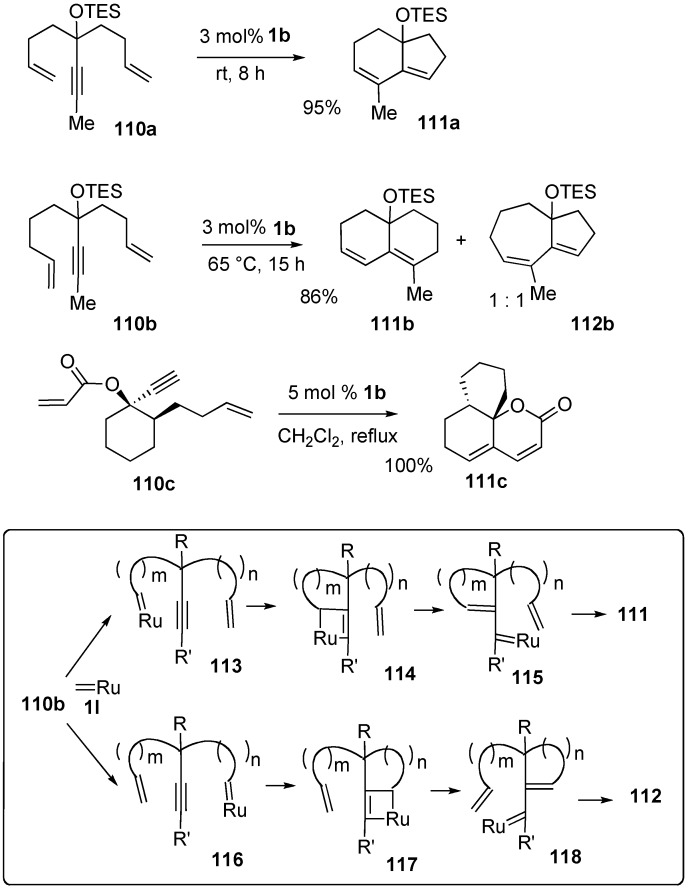
Dienyne metathesis.

Grubbs reported an ingenious method for synthesizing bicyclic compounds from dienynes taking advantage of the metathesis reaction (Scheme 31) [81,82]. When a benzene solution of dienyne **110a** was stirred in the presence of 3 mol % of **1b**, bicyclic compound **111a** was obtained in 95% yield in one operation. In the case of **110b**, two bicyclic compounds **111b** and **112b** were formed. Furthermore, dienyne **110c** gave tricyclic compound **111c** in a quantitative yield. Probably, ruthenium carbene methylidene complex **1l** reacts with an alkene moiety in dienyne **110b** to give ruthenium carbene complex **113** and/or **116**, which reacts with an alkyne part to give ruthenacyclobutene **114** and/or **117**. Ring opening of this complex gives ruthenium carbene complex **115** and/or **118**, which reacts with the alkene part intramolecularly to give ruthenacyclobutane. Ring opening of this complex gives bicyclic compounds, **111** and/or **112**.

**Scheme 32:**
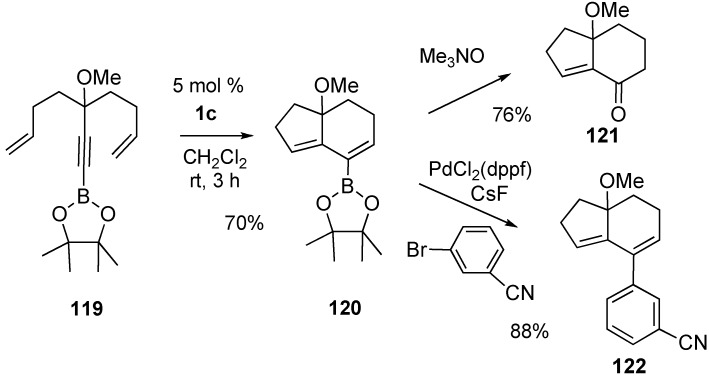
Reaction of eneyne having alkynyl boronate.

The transformation of dienyne into the bicyclic vinylboronate is shown in Scheme 32. Alkynylboronate **119** cleanly underwent ruthenium-promoted metathesis in 70% yield. The resultant bicyclic dialkenylboronic ester **120** was efficiently oxidized to corresponding enoate **121** by treatment with Me_3_NO in refluxing THF. In the presence of CsF, reaction of **120** with 3-bromobenzonitrile under the catalysis of PdCl_2_(dppf) furnished cross-coupling product **122** (Scheme 32) [83].

**Scheme 33:**
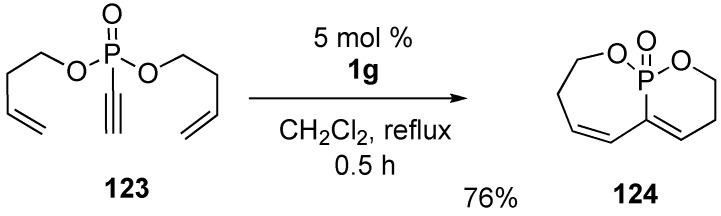
Synthesis of phosphorous mono- and bicycles by RCM.

A versatile route for the synthesis of a phosphorus oxide template was presented (Scheme 33). Dienyne metathesis using **1g** on substrate **123** led to the formation of bicyclic phosphorus heterocycles **124** [84].

**Scheme 34:**
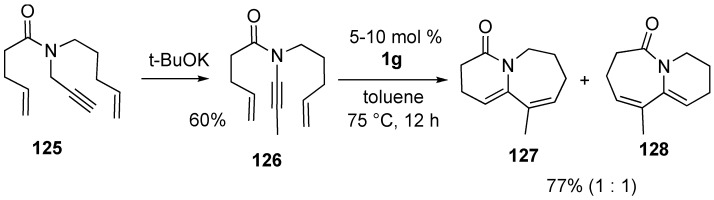
Base-promoted isomerization of propargyl amide followed by RCM.

Base-promoted isomerization of propargyl amide **125** gave alkynyl amide **126**, dienyne metathesis of which gave bicyclic compounds **127** and **128** in a ratio of 1:1 (Scheme 34) [85].

**Scheme 35:**
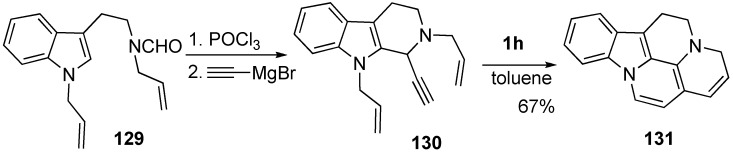
Dienyne metathesis of β-carboline derivative.

Dienyne metathesis of β-carboline derivative **130** afforded oxidized pentacyclic compound **131** related to alkaloids containing a β-carboline unit. The starting material **129** was readily synthesized from tryptamine derivative (Scheme 35) [86].

### Syntheses of Natural Products and Related Compounds Using Dienyne Metathesis

Dienyne metathesis is a useful method for the synthesis of fused bicyclic or polycyclic compounds in one step, and many bond fissions and bond formations occur during the dienyne metathesis. Therefore, when dienyne metathesis is used for the total synthesis of natural products, retro synthetic analysis for them is unique, and the reaction process is generally shortened.

**Scheme 36:**
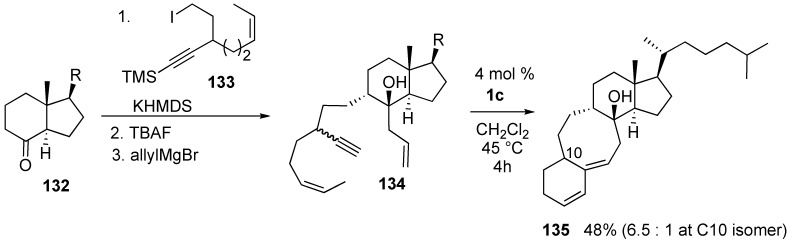
Synthesis of a [6.4.0]carbocyclic system.

A new approach to the synthesis of a linearly fused 6–8-6 tricarbocyclic ring system was realized using dienyne metathesis [87]. This ring system is a carbon framework analogous to the proposed transition state of isomerization of previtamin D_3_ to vitamin D_3_. The starting dienyne **134**, which was prepared by condensation of indenone **132** and alkyne **133** followed by deprotection and then introduction of an allyl group, was reacted with **1c** to give target molecule **135** as a diastereomeric mixture at the C-10 position in 48% yield (Scheme 36).

**Scheme 37:**
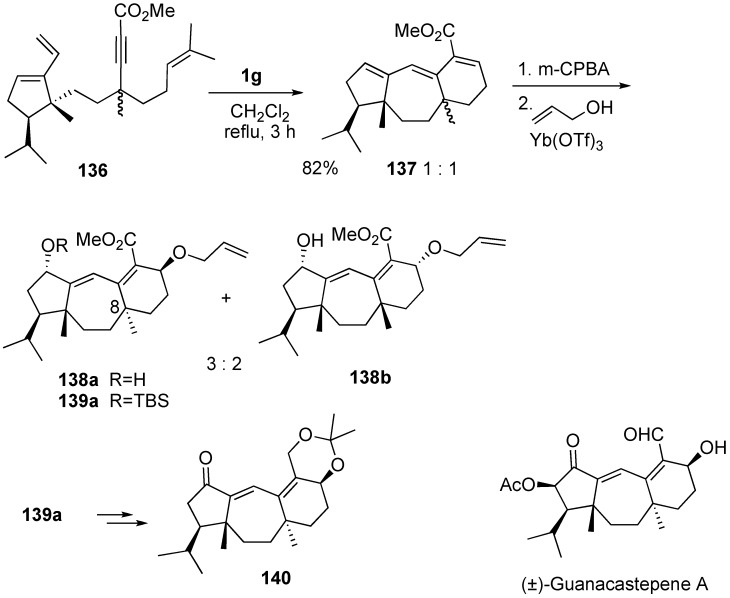
Synthesis of (±)-guanacastephene A.

A concise route to a key intermediate in the total synthesis of guanacastepene A using dienyne metathesis was reported [88]. The main feature includes the construction of fused seven- and six-membered rings. Metathesis of dienyne **136** was carried out using **1g** and a mixture of tricyclic compounds **137** was obtained in a ratio of 1 to 1. Selective epoxidation followed by introduction of an allyloxy group in the presence of Yb(OTf)_3_ gave **138a** and **138b**. Protection of the hydroxyl group of **138a** gave **139a**, and then it was led to compound **140**, which was already converted into (±)-guanacastepene A (Scheme 37).

**Scheme 38:**
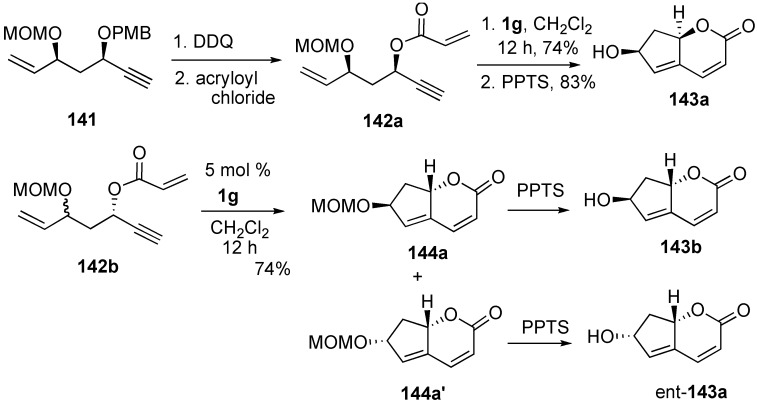
Synthetic study directed toward the total synthesis of ilexlactone.

Krishna reported the synthetic study toward the stereoselective total synthesis of ilexlactone, the structure of which was already assigned as **143a**. Bicyclic ring system was constructed from dienyne **142a** using 5 mol % of **1g** in 74% yield. Deprotection of the MOM group afforded the target molecule **143a**. However, the spectral data of this compound was found to be different from those of ilexlactone reported in the literature. Thus, the authors further synthesized ent-**143a** and **143b** from **142b** in a similar procedure. However, the spectral data of ent-**143a** and **143b** were found to be different from those of ilexlactone. Thus, the authors concluded that the structure proposed ilexlactone was incorrect (Scheme 38) [89].

**Scheme 39:**
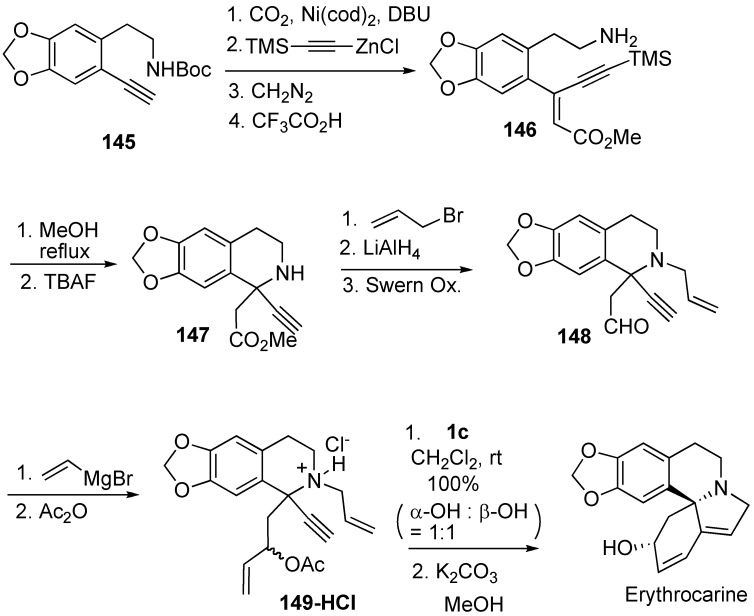
Total synthesis of erythrocarine.

Total synthesis of (±)-erythrocarine was achieved by Mori using dienyne metathesis [90]. Synthesis of trisubstituted alkene **146** was achieved with a regio- and stereoselective introduction of carbon dioxide and an alkynyl group onto the terminal alkyne of **145** followed by desilylation. Hetero-Michael reaction of **146** gave isoquinoline derivative **147**, which was converted into dienyne **149**. Since the tertiary amine of **149** coordinates to the ruthenium catalyst and the catalytic activity is decreased, dienyne metathesis of **149·HCl** was carried out using ruthenium catalyst **1c** to give tetracyclic compounds as a diastereomeric mixture in a ratio of 1:1 in quantitative yield. Deacetoxylation of α-isomer gave erythrocarine (Scheme 39). Hatakeyama succeeded in total synthesis of erythravine using a similar procedure [91].

**Scheme 40:**
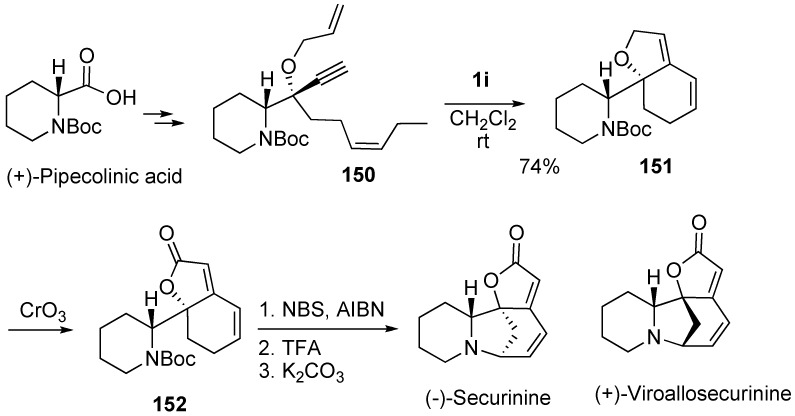
Total synthesis of (-)-securinine.

Honda *et al.* succeeded in a diastereoselective total synthesis of (-)-securinine in an optically pure form by employing RCM of the corresponding dienyne **150** as a key step [92]. They synthesized dienyne **150** having terminal alkene and disubstituted alkene parts from (+)-pipecolinic acid, because ruthenium-carbene complex would at first react with the terminal alkene to form a furan ring. Thus, dienyne metathesis of **150** was carried out using **1i** [18] to give bicyclic compound **151** in good yield. Oxidation of **151** with CrO_3_ gave lactone **152**, which was treated with NBS and then TFA to produce (-)-securinine (Scheme 40). They also synthesized viroallosecurinine in a similar manner [93].

**Scheme 41:**
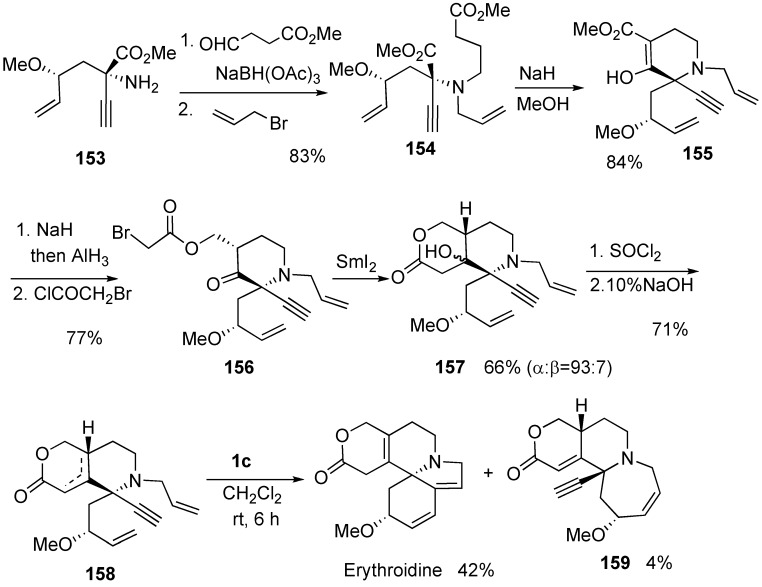
Total synthesis of (+)--erythroidine.

Hatakeyama succeeded in the total synthesis of erythroidine [94]. Construction of C and D rings was begun from amino acid **153**, which was led to dienyne **158**. Dienyne metathesis was carried out using **1c** to give erythroidine in 42 % yield along with compound **159** in 4% yield. In this case, **1g** turned out to be less effective and gave erythroidine in less than 30% yield (Scheme 41).

**Scheme 42:**
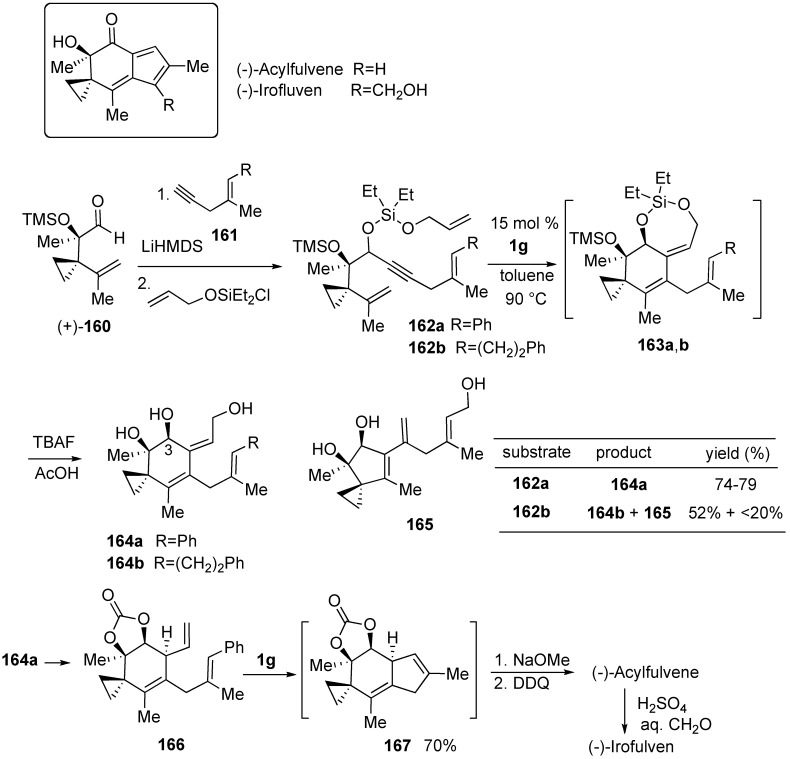
Synthesis of (-)-acylfulvene and (-)-irofulven.

The successful enantioselective syntheses of (-)-acylfulvene and (-)-irofulven was achieved by use of ring-closing metathesis strategy [95]. Synthesis of these compounds began through coupling of readily available aldehyde (+)-**160** and enyne **161**. Treatment of **162a** with **1g** catalyst (15 mol %) in toluene at 90 °C afforded desired **163a** in 90% yield, which was readily converted into the key triol **164a** (3S/3R = 6:1) after desilylation. When trienyne **162b** was treated in a similar manner, desired **164b** was obtained in 52% yield along with minor by-product **165** in 20% yield. The key triol **164a** was converted into triene **166**. Diene metathesis of **166** using **1g** (15 mol %) proceeded smoothly to give bicyclic compound **167**, crude product of which was directly converted into (-)-acylfulvene. According to the known method, (-)-acylfulvene was converted into (-)-irofulven (Scheme 42).

**Scheme 43:**
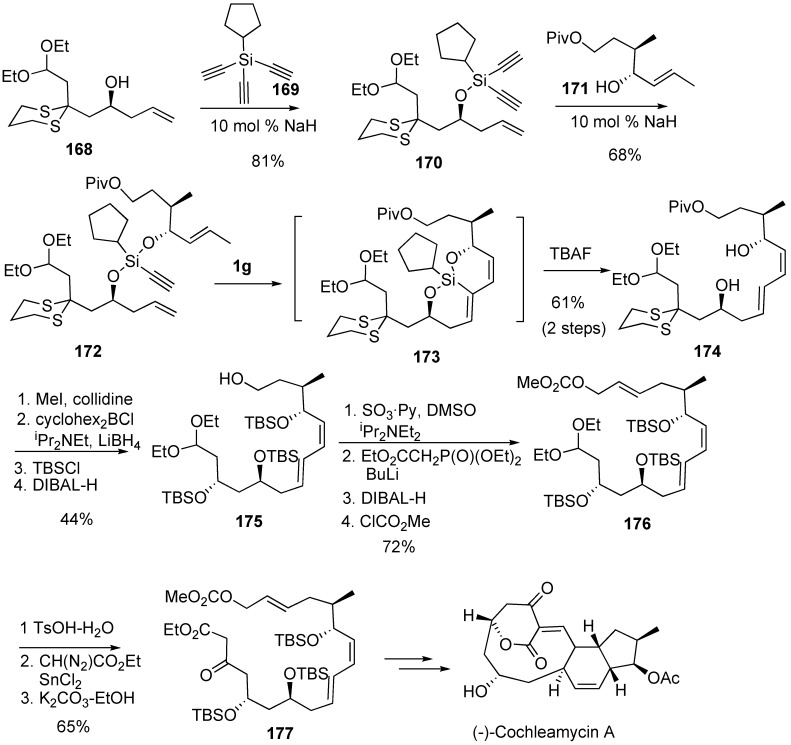
Formal total synthesis of (-)-cochleamycin A using dienyne metathesis.

For the synthesis of (-)-cochleamycin, conjugate diene **174** was synthesized from **172** by dienyne metathesis as a key step. The reaction of alcohol **168** with trialkyne **169** gave alcohol **170**, which was further reacted with alcohol **171** to give dienyne **172**. A tandem ring-closing metathesis of dienyne substrate **172** proceeded to provide a bicyclic siloxane **173**. Removal of the silicon tether of **173** afforded an (*E,Z*)-1,3-dienediol **174**, which was converted into the key compound **177** for the synthesis of (-)-cochleamycin (Scheme 43) [96].

## 3. Cross Enyne Metathesis

**Scheme 44:**
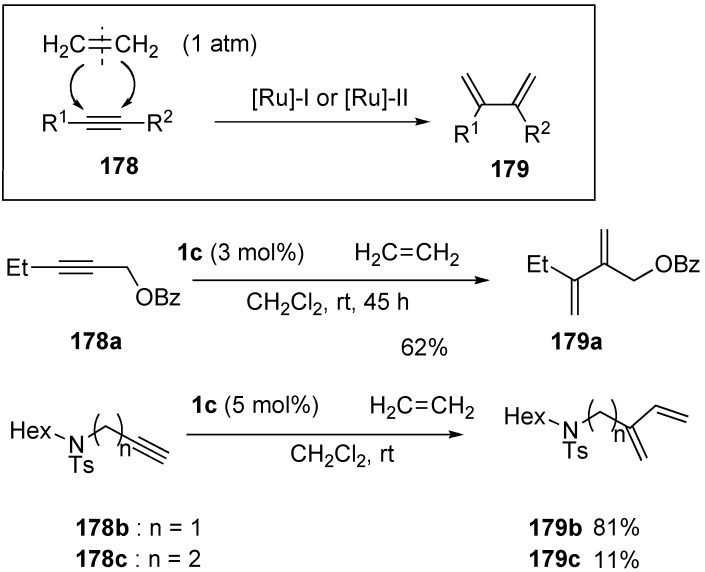
Synthesis of 1,3-diene using cross metathesis.

**Scheme 45:**
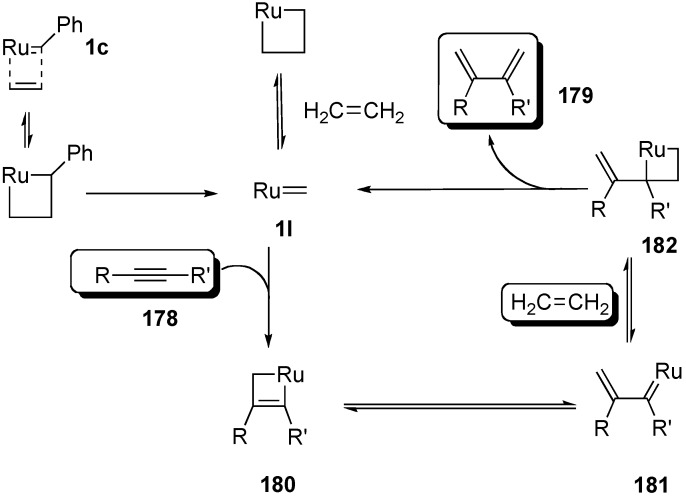
Possible reaction course for formation of 1,3-diene.

A novel synthetic procedure of 1,3-diene from alkyne and ethylene using cross enyne metathesis was developed in 1997 by Mori [97,98]. When a CH_2_Cl_2_ solution of alkyne **178a** was stirred under an atmosphere of ethylene at room temperature in the presence of **1c**, 1,3-diene **179a** was obtained in 62% yield. It is interesting that, formally, the double bond of ethylene is cleaved and each methylene part of ethylene is introduced onto the alkyne carbon to produce 1,3-diene **179** (Scheme 44). The possible reaction course was shown in Scheme 45. Reaction of ruthenium carbene methylidene complex **1l**, generated from **1c** and ethylene, with alkyne **178** gives ruthenacyclobutene **180**, ring-opening of which gives ruthenium carbene **181**. It reacts with ethylene to afford ruthenacyclobutane **182**, ring-opening of which gives 1,3-diene **179**, and **1l** is reproduced. If **1l** reacts with ethylene, this is non-productive process and **1l** would be reproduced. However, this method has a problem, that is: propargyl ester **178a** or amide **178b** gave good results, while homopropargyl amide **178c** led to 1,3-diene **179c** in only 11% yield (Scheme 44). Presumably, a heteroatom at the propargylic position is important, and the ruthenium catalyst would be coordinated by the heteroatom at first and then the reaction proceeds.

When the second-generation ruthenium-carbene complex **1g** was used for this reaction, an alkyne **178** lacking heteroatoms at the propargylic positions, gave 1,3-dienes **179** in good yield (Table 1) [99,100,101,102,103]. Furthermore, the reaction was more rapid and the functional groups on the alkyne were tolerated.

**Scheme 46:**
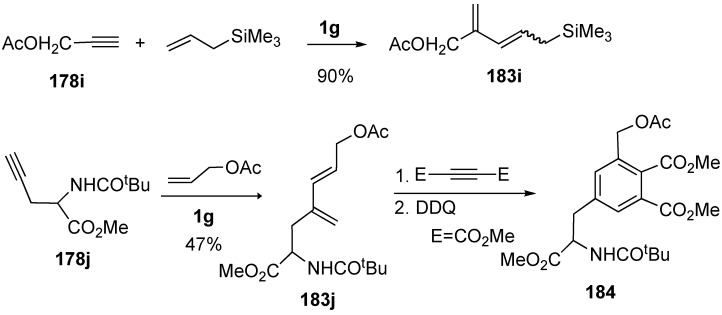
Synthesis of alanine derivative.

Cross metathesis between terminal alkynes **178i** and terminal alkenes was subsequently developed by Blechert, and 1,3-disubstituted diene **183i** was obtained in high yield [104]. Phenylalanine derivative **184** could be synthesized by use of this procedure followed by Diels-Alder reaction (Scheme 46) [104,105].

**Table 1:** Synthesis of Various 1,3-dienes from alkyne and alkene using **1g**.^a^

entry	alkyne **178**	1,3-diene **179**	time (h)	yield (%)
1	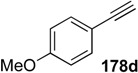	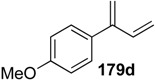	0.5	88
2	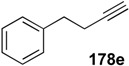	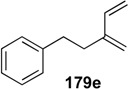	0.5	71
3	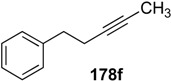	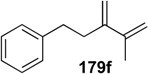	0.5	85
4	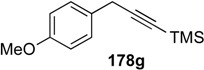	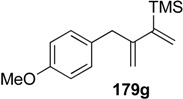	16	87
5	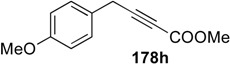	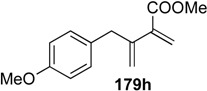	16	43

^a^ All reactions were carried out using 5 mol % of 1 g under 1 atm. pressure of ethylene gas in toluene at 80 °C. ^b^ The starting material was recovered in 10% (entry 4) and 34% (entry 5) yields, respectively.

**Scheme 47:**
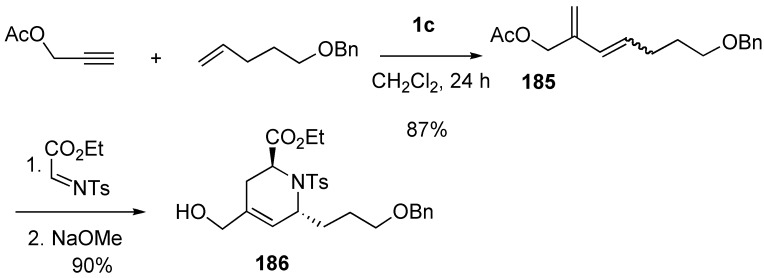
One-step synthesis of pipecolinic acid.

A short and efficient synthesis of highly substituted tetrahydropyridines was achieved from a monosubstituted alkyne, a terminal alkene, and an imine by a combination of cross enyne metathesis and aza-Diels-Alder reaction under high pressure. Cross metathesis of terminal alkyne and alkene afforded diene **185**, which was reacted with imine to give pipecolinic acid derivative **186** in high yield (Scheme 47) [106].

**Scheme 48:**
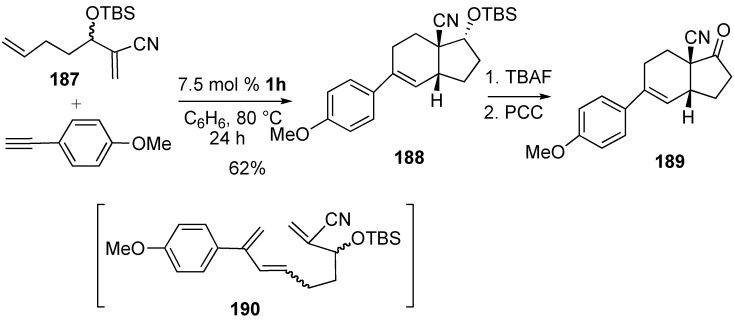
Synthesis of cic-fused carbo-bicycles.

The reaction was further extended to intramolecular Diels-Alder reaction, and cis-hexahydro-1*H*-indene **189** was synthesized from diene **187** and terminal alkyne in one operation [107]. The intermediate would be **190**, which was spontaneously converted into **188**. Deprotection of the silyl group followed by PCC oxidation gave indanone **189** (Scheme 48).

**Scheme 49:**
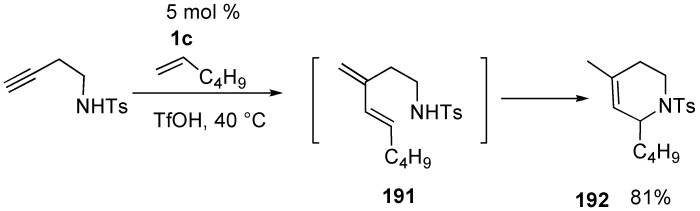
One pot synthesis of nitrogen heterocycles using cross metathesis.

One pot synthesis of nitrogen and oxygen heterocycles was reported using intermolecular cross enyne metathesis in the presence of Bronsted acid (Scheme 49) [108].

**Scheme 50:**
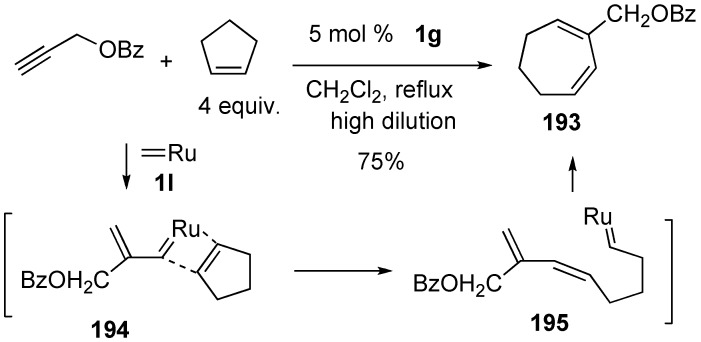
Cross metathesis of cyclopentene and alkyne.

Diver reported cross metathesis of terminal alkyne and cyclopentene using **1g** [109]. Ruthenium-carbene complex **1l** reacts with the terminal alkyne to produce ruthenium-carbene complex **194**, which reacts with cyclopentene to produce ruthenium-carbene complex **195**. It reacts with the alkene part to afford a cycloheptadiene derivative **193** (Scheme 50).

### Synthesis of Natural Products and Related Compounds Using Cross Metathesis

**Scheme 51:**
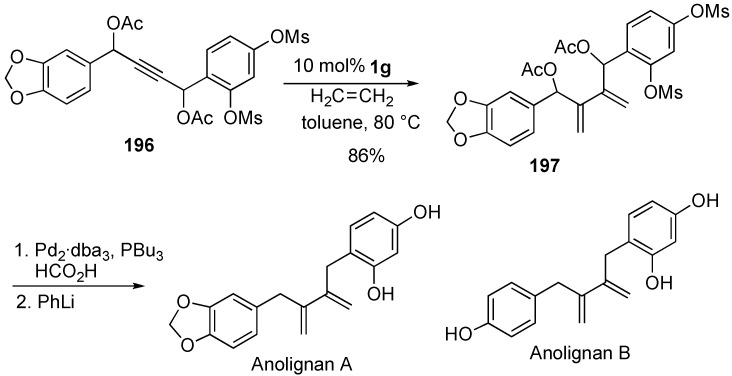
Synthesis of anolignan A using cross enyne metathesis.

Synthesis of natural products using cross metathesis is interesting because 1,3-diene moiety is constructed onto the alkyne carbons at the later step. Anolignans were synthesized using cross metathesis of enyne as a key step. 1,3-Diene **197** could be synthesized from alkyne **196** by treatment with **1g** under ethylene gas. Palladium-catalyzed deacetoxylation followed by deprotection gave anolignan A. Anolignan B could be synthesized in a similar manner. It was interesting that two methylene parts of the anolignan skeleton could be introduced at a later stage of the total synthesis using cross metathesis (Scheme 51) [110].

**Scheme 52:**
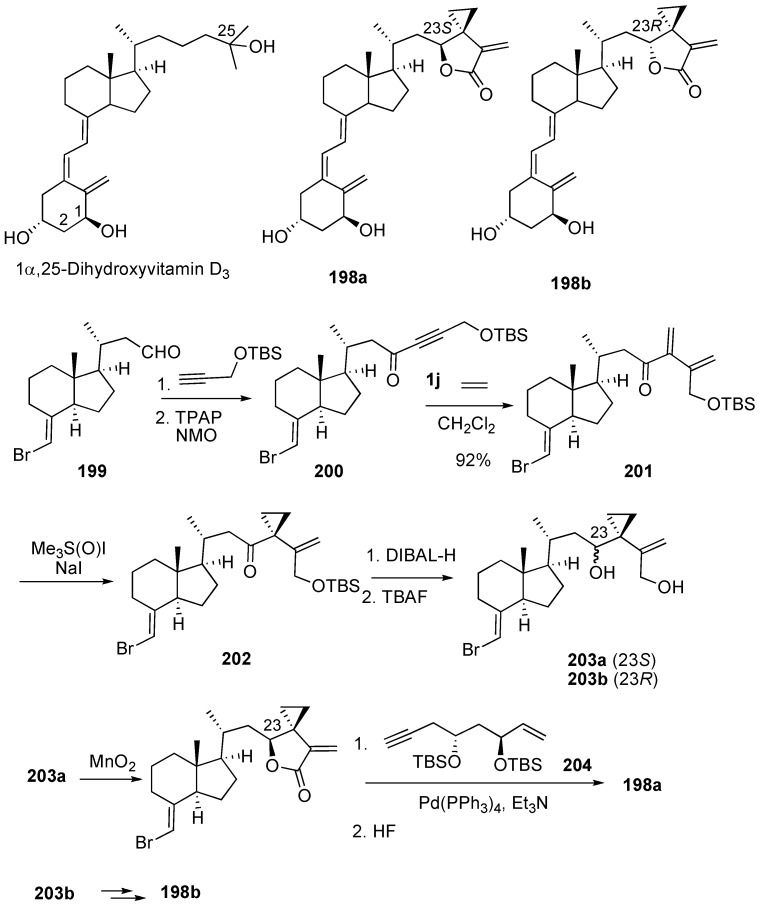
Synthesis of 24,25-ethanovitamine D3 lactones.

Novel vitamin D receptor antagonists, 24,24-ethanovitamine D3–26,23-lactones **198a** and **198b** and their analogs were synthesized (Scheme 52) [111]. The CD-ring precursors **203a** and **203b** were efficiently synthesized *via* ruthenium-catalyzed intermolecular enyne metathesis of **200** with ethylene as a key step. Cyclopropanation of resultant enyne metathesis product **201** followed by treatment with DIBAL-H and then deprotection gave compounds **203a** and **203b**. Oxidation of **203a** and **203b** followed by palladium-catalyzed coupling reaction with **204** and then deprotection afforded **198a** and **198b**, respectively.

**Scheme 53:**
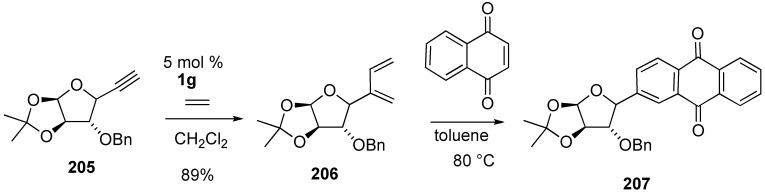
Synthesis of C-aryl glycoside.

A versatile strategy for the synthesis of C-aryl glycoside was successfully developed. An intermolecular enyne metathesis of C-alkynyl glycoside **205** with ethylene gave diene **206**, which was followed by Diels Alder reaction and then aromatization to provide C-aryl glycoside **207** (Scheme 53) [112].

**Scheme 54:**
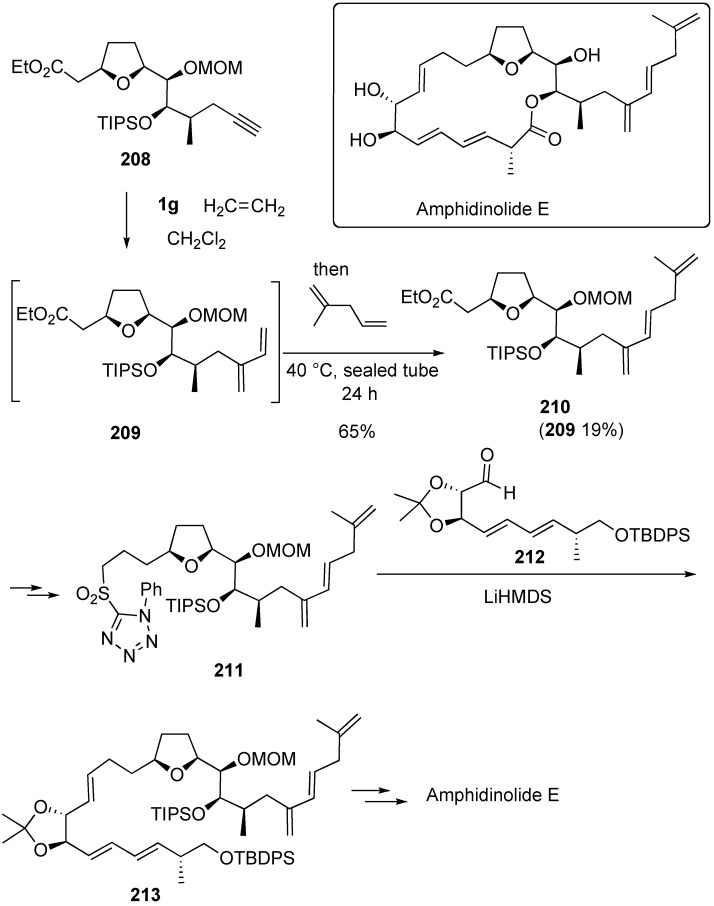
Synthesis of amphidinolide E.

Lee succeeded in the total synthesis of (-)-amphidinolide E, whose side chain was constructed using cross enyne metathesis [113,114]. Alkyne **208** was first reacted with ethylene in the presence of **1g** to give **209**, which was further engaged *in situ* in a chemoselective cross metathesis with 2-methyl-1,4-pentadiene to give triene **210** in 65% yield along with diene **209** in 19% yield. Isolated diene **209** was further recycled and reacted with 2-methyl-1,4-pentadiene in the presence of **1g** to afford triene **210**, which was further elaborated into **211**. Condensation of **211** and **212** afforded compound **213**. From this compound **213**, total synthesis of amphidinolide E was achieved (Scheme 54).

Recently, same group succeeded in the total synthesis of amphidinolide K using intramolecular cross enyne metathesis in the key step [115].

**Scheme 55:**
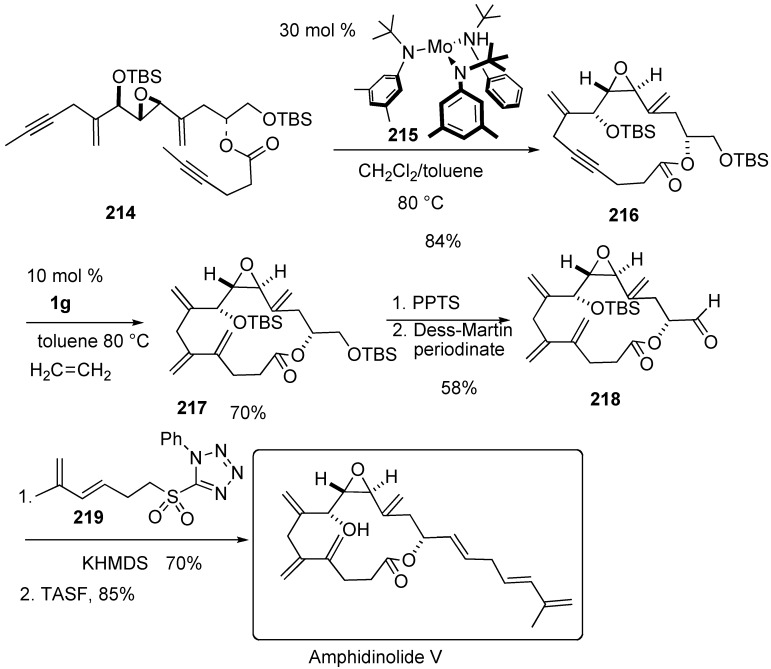
Synthesis of amphidinolide V.

Fürstner succeeded in the total synthesis of amphidinolide V using ring-closing alkyne metathesis for the construction of the macrocycle (Scheme 55). Dialkyne **214** was treated with molybdenum complex **215** to give macrocycle **216** in 84% yield. Then an intermolecular enyne metathesis of the resulting cycloalkyne **216** with ethene was used to set the vicinal exo-methylene branches. From this compound **217**, amphidinolide V was synthesized. As the result, the absolute configuration of amphidinolide V was determined to be as 8*R*, 9*S*, 10*S*, 13*R* [116,117].

### 4. Ring-Opening Enyne Metathesis

**Scheme 56:**
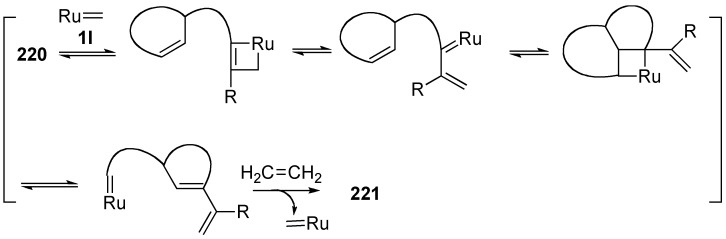
Possible reaction course for ROM of **220**.

Tandem metathesis of cycloalkene-ynes is a unique reaction because different cyclic compound is formed from the starting cycloalkene *via* many steps by a one operation. These reactions are contained ring opening metathesis (ROM), ring closing metathesis (RCM) and/or cross metatheses (CM). Mori reported the ROM of cycloalkene-yne [118]. When cycloheptene-yne **220a**, the substituent of which was placed at the 3-position of cycloalkene, was reacted with the first generation ruthenium carbene complex **1c** in CH_2_Cl_2_ under ethylene gas at room temperature for 24 h, pyrrolidine derivative **221a** was obtained in 56% yield (Table 2, entry 1). Various cycloalkene-ynes **220** gave pyrrolidine derivatives **221** in high yields by a one-pot operation. Formally, in this reaction, the double bonds of ethylene and cycloalkene were cleaved and each methylene part of ethylene was introduced onto the alkyne and cycloalkene carbons, respectively, and a carbon-carbon double bond was formed between the alkyne and cycloalkene carbons to form a pyrrolidine ring (Figure 3). In each case, pyrrolidine ring is formed and the length of the substituent corresponds to the initial ring size minus 1. The possible reaction course for formation of **221** from **220** was shown in Scheme 56.

**Table 2:** ROM-RCM of cydoalkene-yne. 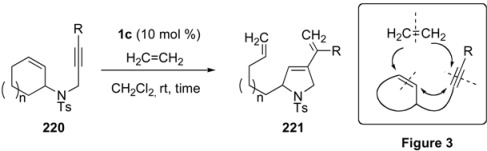

entry	R	ring size	N	time (h)	yield (%)*^a^*
1	**220a** Me	7	2	24	**221a** 56*^b^*
2	**220b** H	6	1	4	**221b** 78
3	**220c** H	7	2	1	**221c** 70
4	**220d** H	8	3	1	**221d** 75

^a^ Yields were calculated from 1H NMR. ^b^ **220a** was recovered in 36% yield.

**Scheme 57:**
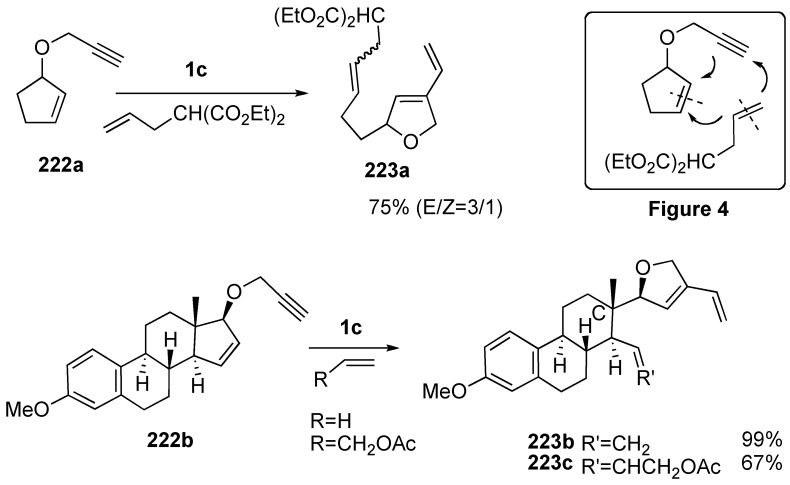
ROM-CM enyne in the presence of alkene.

Blechert reported the same type of ROM-CM. Reaction of cyclopentene derivative **222a** having a propargyloxy group at the 3-position with **1c** in the presence of diethyl allyl malonate afforded compound **223a** in 75% yield [119,120]. In this reaction, the cleaved alkylidene part of diethyl allyl malonate is introduced onto the cyclopentene carbon, and the methylene part is introduced onto the alkyne carbon to form furan derivative **223a** (Figure 4). Furthermore, the five-membered ring of estrone **222b** or **222c** is cleaved using **1c** in the presence of an alkene to give **223b** or **223c**, and an alkene part is introduced onto the C-ring (Scheme 57).

**Scheme 58:**
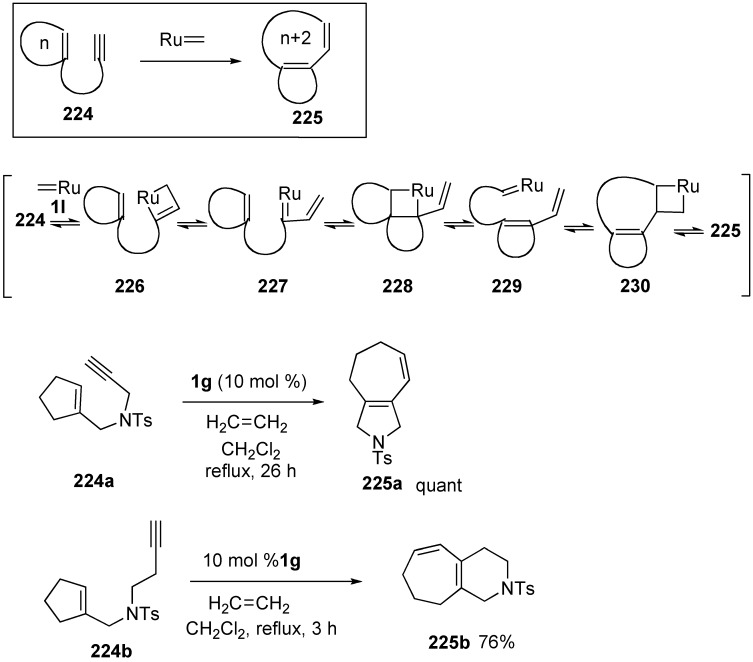
Ring-opening metathesis of cycloalkene-yne.

Ring-opening metathesis of 1-substituted cycloalkene-yne **224** with **1g** afforded bicyclic compound **225** [121,122]. Reaction of an alkyne part of **224** with ruthenium carbene complex **1l** gives ruthenacyclobutene **226**. Ring opening of this gives ruthenium carbene complex **227**, which reacts with cycloalkene to afford highly strained ruthenacyclobutane **228**. Ring opening of this affords ruthenium carbene complex **229** and then it reacts with alkene part to afford ruthenacyclobutane **230**, ring-opening of which gives bicyclic compound **225**. When a CH_2_Cl_2_ solution of cyclopentene derivatives **224a** was stirred in the presence of **1g** under ethylene gas for 26 h, bicyclic compound **225a** was obtained in quantitative yield. In a similar manner, cyclopentene-yne **224b**, the side chain of which was elongated, was reacted with **1g** to give bicyclic compound **225b** in 76% yield. These results indicated that the initial ring (n) was enlarged to (n + 2) ring and the size of the other ring corresponds to the carbon chain length from an alkyne carbon to an alkene carbon (Scheme 58).

**Scheme 59:**
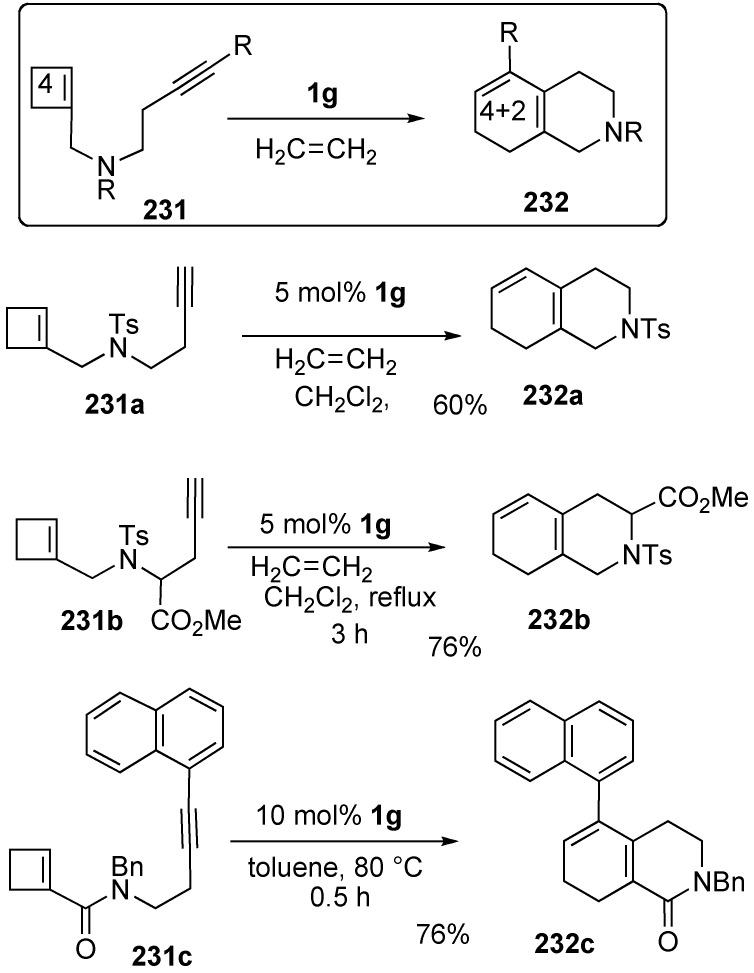
Synthesis of isoquinoline derivatives using ROM of cyclobutene-yne.

To synthesize an isoquinoline derivative using this method, the initial cycloalkene would be cyclobutene and the chain length between alkyne and alkene carbons containing nitrogen would be four. Treatment of cyclobutene-yne **231a** with **1g** afforded isoquinoline derivative **232a** in 60% yield in one step. Furthermore, glycine derivative **231b** having a cyclobutene ring in a tether afforded cyclic amino acid **232b** in 76% yield. This procedure was further extended to the synthesis of biaryl compound **232c** from cyclobutene-yne **231c**. It was interesting that in this case, an aryl group on the alkyne of **231c** is placed at the 5-position of isoquinoline **232c** (Scheme 59) [123].

**Scheme 60:**
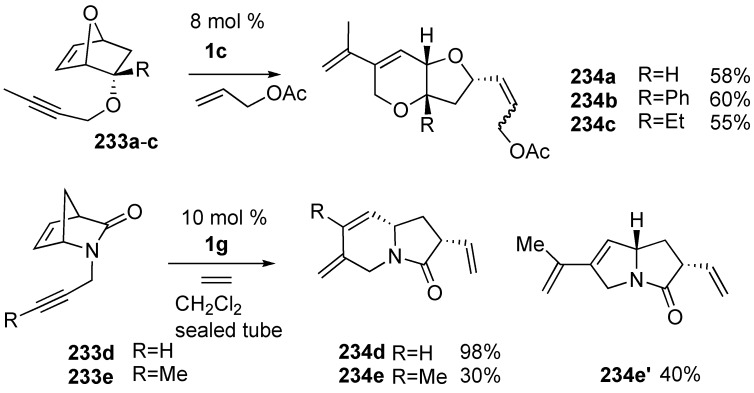
ROM-RCM followed by CM of cycloalkene-yne.

Plumet *et al.* described domino metathesis of propargyl (2-endo-7-oxanorborn-5-enyl) ethers **233a**–**c** with allyl acetate in the presence of Grubbs’ ruthenium catalyst **1c** (Scheme 60) [124,125]. The reaction proceeded stereoselectively to produce substituted *cis*-fused bicyclic ethers **234a**-**c**. In a similar manner, indolizidinone derivative **234d** was obtained from azabicyclo[2.2.1]heptenone **233d** in high yield. Later, the substituent effect of this reaction was further investigated and pyrrolizidinone derivative **234e** was obtained in 40% yield along with indolizidinone derivative **234e’** in 30% yield when the toluene solution of **233e** (R=Me) and **1g** was warmed at 80 °C for 30 min under ethylene gas [126].

**Scheme 61:**
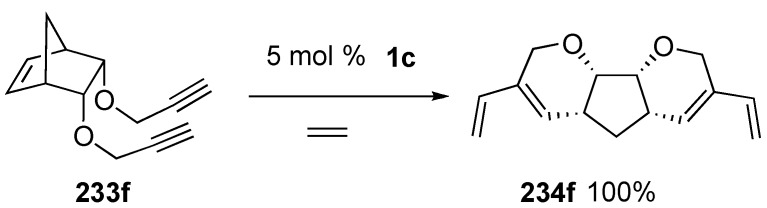
ROM-RCM of norbornene derivative.

North and Banti observed double ring-opening metathesis of dialkynyl cycloalkenes **233f** affording tricyclic compound **234f** in high yield (Scheme 61) [127,128].

## 5. Skeletal Reorganization Using Transition Metals

**Scheme 62:**
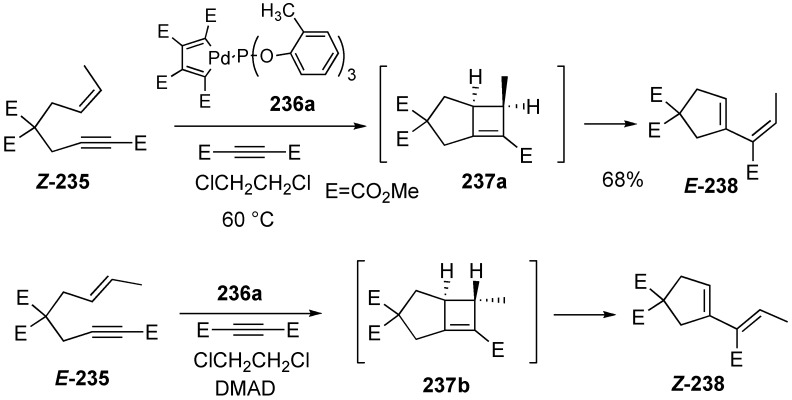
Enyne metathesis using palladium catalyst.

Trost discovered palladium-catalyzed enyne metathesis during the course of his study on palladium-catalyzed enyne cyclization [129,130,131,132,133,134,135]. Treatment of Z-**235** with palladacyclopentadiene (TCPT, **236a**) in the presence dimethyl acetylene dicarboxylate (DMAD) in dichloroethane at 60 °C led to metathesis product *E*-**238** in 68 % yield, which consisted of only *E*-isomer *E*-**238**. Similarly, the *E*-substrate *E*-**235** gave predominantly *Z*-**238** (Scheme 62) [129].

**Scheme 63:**
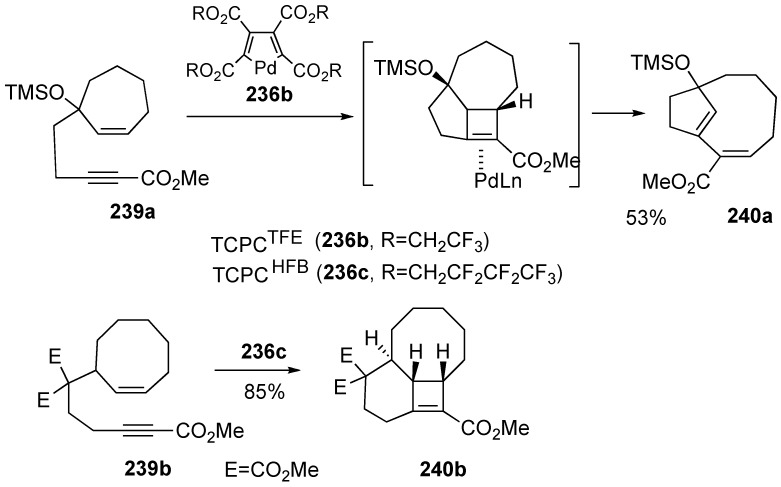
Confirmation of four-membered ring.

This method provides a very simple route to bridged bicycles possessing bridgehead olefins (Scheme 63). When enyne **239a** was treated with TCPC^TFE^ **236b**, bicyclo[6.2.1]undecadiene **240a** was formed in 53% yield. When a mixture of 4% TCPC^HFB^ **236c**, 4% tri-*o*-tolylphosphate, bis(heptafluorobutyl)-acetylenedicarboxylate and enyne **239b** in dichloroethane was heated at 80 °C, tricyclic compound **240b** was obtained in 85% yield. It means that the reaction proceeds *via* the formation of the four-membered-ring [132].

**Scheme 64:**
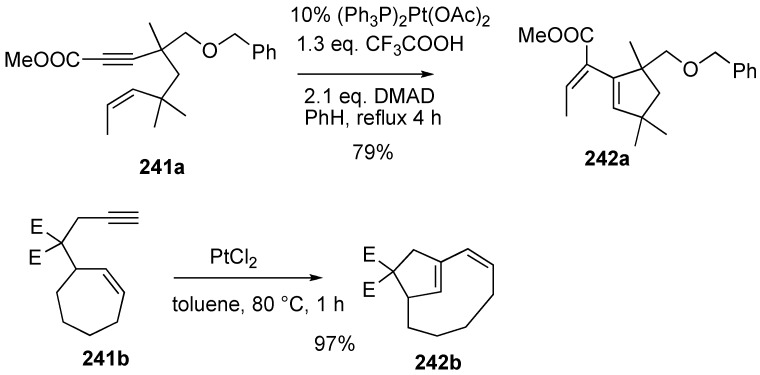
Platinum-catalyzed skeletal reorganization.

The simple platinum complex (Ph_3_P)_2_Pt(OAc)_2_ effected metathesis of enyne. Enyne **241a** gave cyclized compound **242a**, and the yield was comparable to that of TCPC but significantly faster. Murai and Chatani [136] also reported the PtCl_2_-catalyzed reaction of cycloalkene-yne **241b**. In this case, exclusively bicyclic compound **242b** was obtained in 97% yield (Scheme 64).

**Scheme 65:**
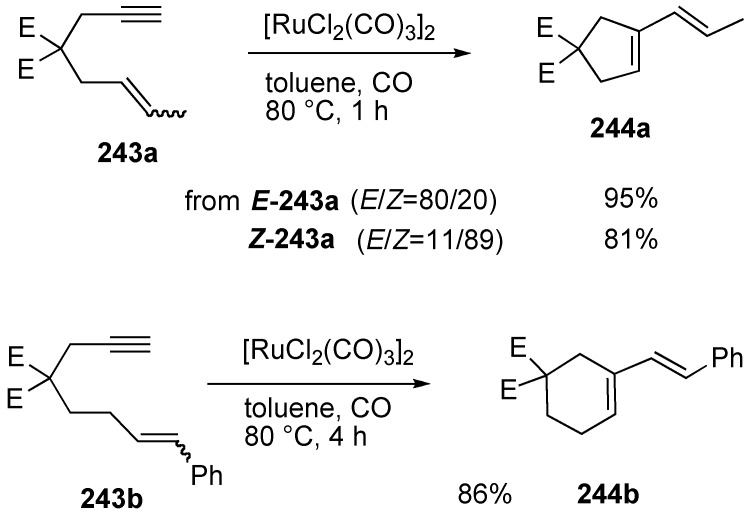
Ruthenium-catalyzed skeletal reorganization.

In 1994, Murai and Chatani reported skeletal reorganization of 1,6-enyne using [RuCl_2_(CO)_3_]_2_ as a catalyst (Scheme 65) [137,138]. When the reaction of ***E*-243a** (*E/Z*=80/20) was carried out in the presence of [RuCl_2_(CO)_3_]_2_ under carbon monoxide, *E*-isomer **244a** was produced predominantly. It is interesting that from *Z*-**243a** (*E/Z*=11/89), *E*-**244a** was formed. An *E/Z* mixture of 1,7-enyne **243b** afforded only *E*-isomer of **244b** in 86% yield.

**Scheme 66:**
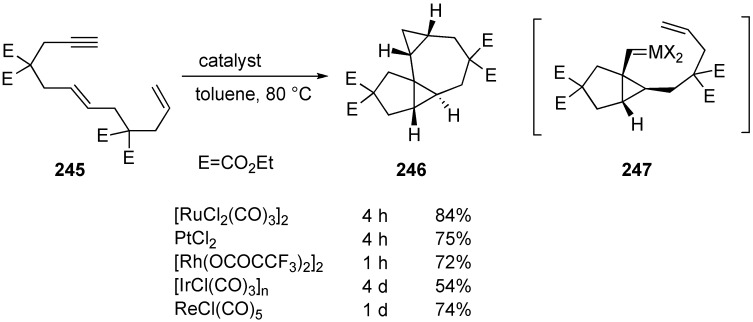
Trapping of carbenoid intermediate.

They speculated that complex **247** having two carbenoid carbons would be generated on the alkyne carbons during the reaction [138]. To trap this intermediate, the reaction of 6,11-dien-1-yne **245**, which has an olefin moiety in a tether, was carried out in the presence of [RuCl_2_(CO)_3_]_2_ in toluene at 80 °C for 4 h to give tetracyclic compound **246** containing two cyclopropane rings in 84% yield. It is interesting to note that other transition-metal complexes, such as PtCl_2_, [Rh(OOCCF_3_)_2_]_2_, [IrCl(CO)_3_]_n_, and ReCl(CO)_5_ also showed catalytic activity for this very complex transformation.

Rh(II)-catalyzed skeletal reorganization of 1,6- and 1,7-enynes through electrophilic activation of alkynes was also reported [139,140].

**Scheme 67:**
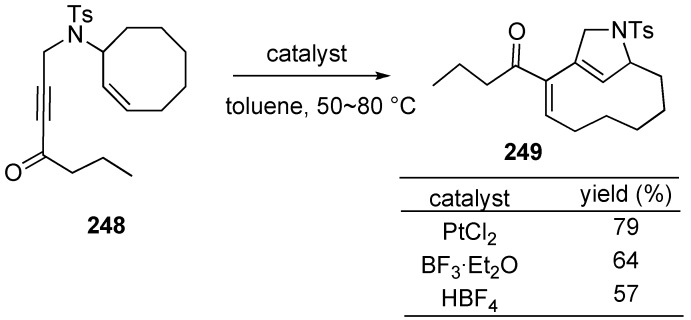
Lewis and Bronsted acids catalyzed skeletal reorganization.

Surprisingly, in some cases, simple Lewis or Brønsted acids as the catalysts could replace PtCl_2_ (Scheme 67). Treatment of **248** with BF_3_·Et_2_O or HBF_4_ gave skeletal reorganization product **249** in good yield [141].

**Scheme 68:**
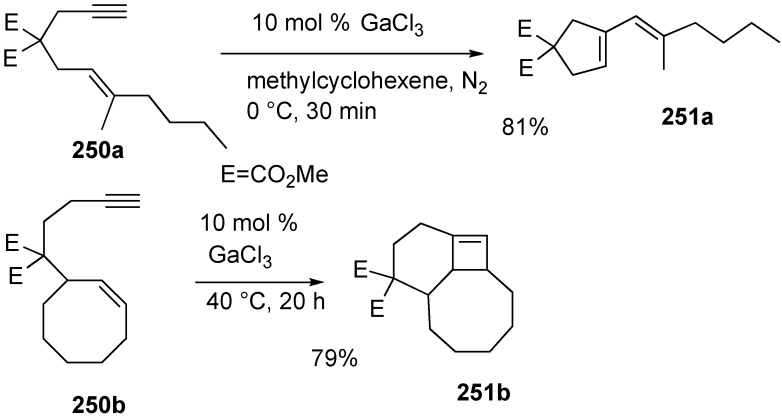
Skeletal reorganization by GaCl3.

Murai and Chatani group reported skeletal reorganization of enynes to 1-vinylcycloalkene by GaCl_3_ [142]. The reaction of **250a** proceeded in methylcyclohexene at 0 °C and was completed within 1 h to give **251a**. It is interesting that highly strained cyclobutene derivative **251b** was obtained from 1,7-enyne **251a** in high yield (Scheme 68).

Presumably, each skeletal reorganization reaction starts by coordination of the metal to the alkyne part, and then the alkene part would attack the cation center of the alkyne coordinated by metals. However, the reaction mechanism is still not clear, and it is thought that each reaction mechanism differs depending on the metal used [143].

### Synthesis of Natural products Using Skeletal Reorganization

**Scheme 69:**
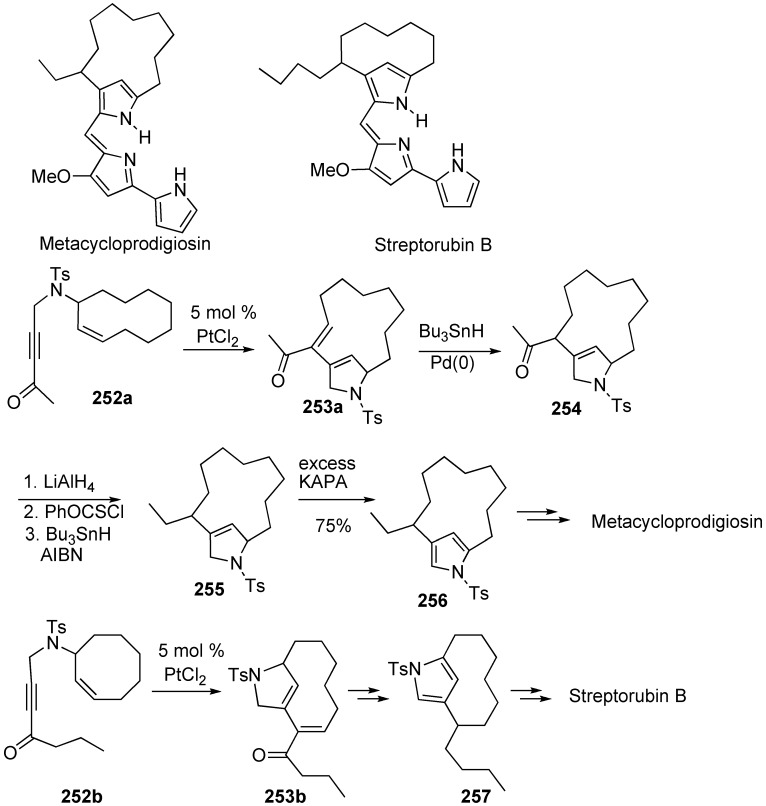
Formal total synthesis of streptorubin B and metacycloprodigiosin.

Skeletal reorganization is a useful tool for the synthesis of complicated natural products. Fürstner achieved formal total syntheses of the antibiotics metacycloprodigiosin and streptorubin B by a platinum-catalyzed skeletal reorganization reaction (Scheme 69) [144]. The key step leading to the meta-bridged pyrrole core structures consisted of a metathesis reaction of electron-deficient enynes **252a** and **252b** catalyzed by PtCl_2_. The skeletal reorganization products **253a** and **253b** were converted into the respective target molecules.

**Scheme 70:**
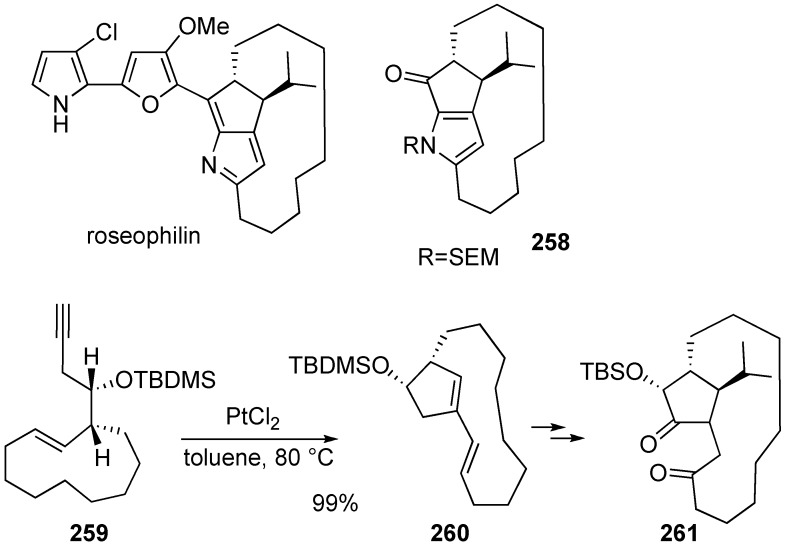
Formal total synthesis of roseophilin.

Trost succeeded in formal total synthesis of roseophilin [145]. Macrocyclic compound **260** was synthesized from enyne **259** by platinum-catalyzed skeletal reorganization. Compound **260** was converted into **261**, which was led to pyrrole derivative **258** and it was already converted into roseophilin (Scheme 70).

## 6. Metallotropic Rearrangement

**Scheme 71:**
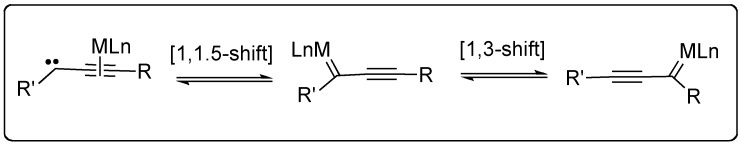
Metalotropic rearrangement.

**Scheme 72:**
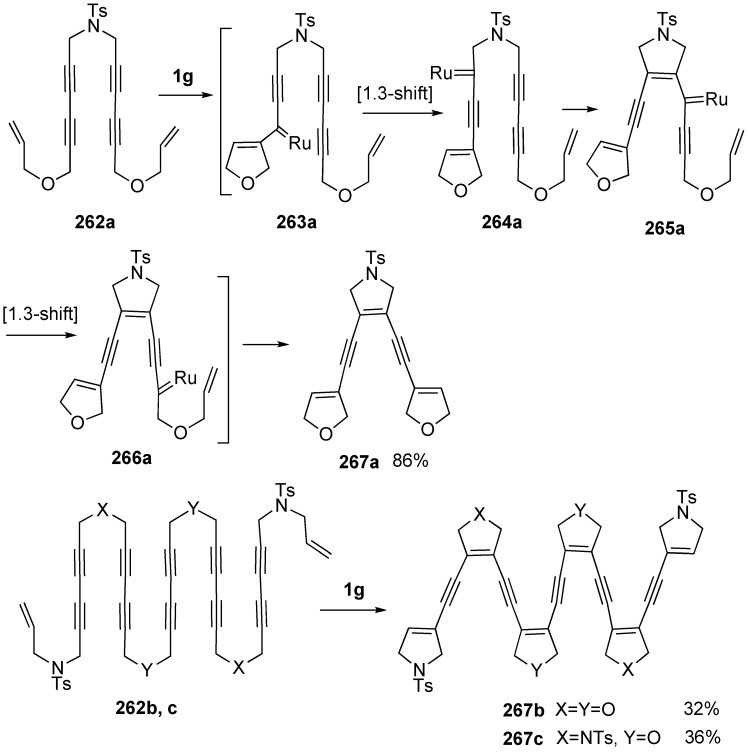
Synthesis of oligoenynes by metathesis of metallotropy.

The bond reorganization processes, defined as metallotropic shift, of various alkynyl carbene complexes with Rh [146], Mn [147], Re [148], Cr [149], Mo [149], and W [149] metals have been already reported. The rearrangement involving Rh, Cr, Mo, and W is a [1,3]-shift, while that with Mn and Re is formally defined as a [1,1.5]-shift. However, the metallotropic shift of ruthenium alkynyl carbene complexes has not been observed until recently (Scheme 71). The metallotropic [1,3]-shift of a transient ruthenium carbene complex is involved in the enyne ring-closing metathesis (RCM) of diyne containing substrates. On the basis of this concept, one-step construction of enediynes and oligoenynes was realized by the uniquely controlled repetitive metallotropic [1,3]-shift of ruthenium carbene species. Reaction of **262a** with **1g** gave ene-diyne **267a** in high yield by one operation. Presumably, reaction of **262a** with **1g** gives ruthenium carbene complex **263a**, which is converted into ruthenium carbene complex **264a** *via* [1,3]-shift. [2+2] Cycloaddition followed by ring opening gives **265a**, which is converted into **266a** *via* [1,3]-shift. From this complex **266a**, ene-diyne **267a** is formed. In a similar manner, **262b** and **262c** gave oligoenynes **267b** and **267c**, respectively, in one operation (Scheme 72) [150].

### Synthesis of Natural Products Using Metallotropic Rearrangement

**Scheme 73:**
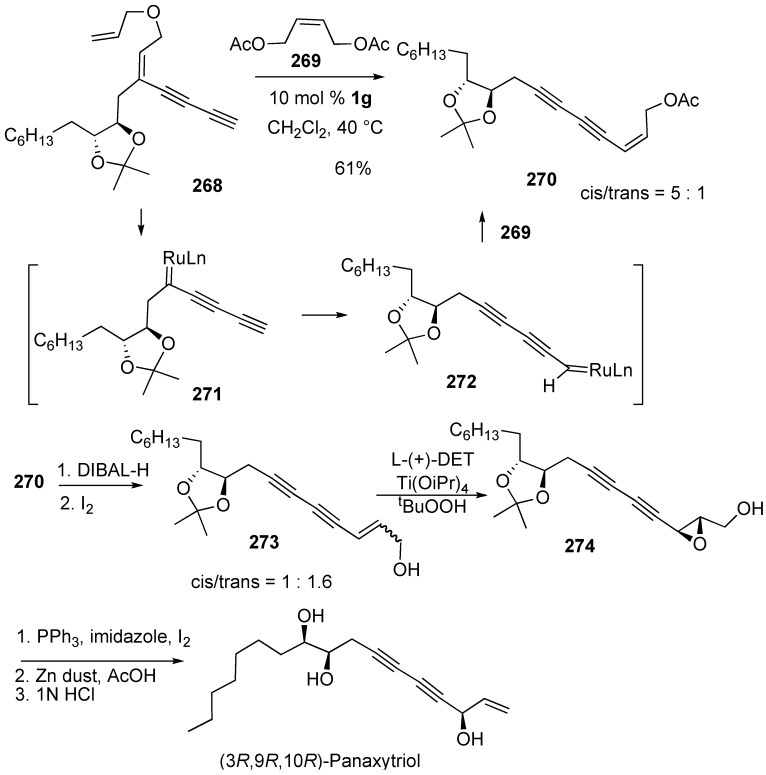
Total synthesis of (3R,9R,10R)-panaxytriol.

The metallotropic [1,3] shift of a transient ruthenium carbene complex is involved in the enyne ring-closing metathesis of diyne containing substrates. Ring-closing metathesis followed by metallotropic [1,3]-shift and cross metathesis allowed for the development of novel strategy for the total synthesis of conjugated 1,3-diyne-containing natural product (3*R*,9*R*,10*R*)-panaxytriol [151]. When compound **268** was treated with **1g** in the presence of 2.0 equiv. of alkene **269**, the expected product **270** was obtained in 61% yield as a mixture of *Z*/*E*-isomers (5:1) together with **268** (10%). Metathesis of diene in **268** gave ruthenium carbene complex **271**, metallotropic [1,3] shift of which gave ruthenium carbene complex **272**. Cross metathesis of **272** and alkene **269** afforded compound **270**. From this compound **270**, (3*R*,9*R*,10*R*)-panaxytriol could be synthesized (Scheme 73).

Total synthesis of (+)-asperpentyn and (-)-tricholomenyn A have been accomplished by implementing this metathesis-based tandem reaction sequence as the key step [152]. Compound **276**, which was prepared from **275** and diyne, was treated with **1g** under ethylene atmosphere gave **279a** and **279b** *via* **277** and **278**. From **279a**, asperpentyn was synthesized. When compound **280** was reacted with ethylene in the presence of **1g**, the reaction did not proceed. In a similar method, compound **281** was treated with **1g** under ethylene to give **282**, which was led to (+)-tricholomenyn.

**Scheme 74:**
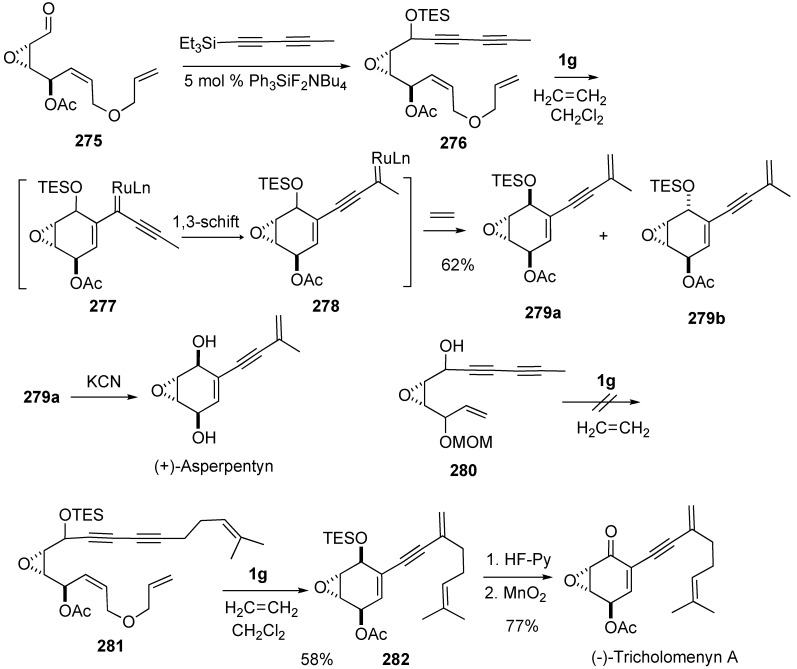
Synthesis of (+)-asperpentyn and (+)-tricholomenyn A using tandem enyne metathsis.

## Perspective

Since the discovery of stable and isolable catalysts for metathesis by Schrock and Grubbs, a wide range of olefin metatheses have been reported, and olefin metathesis now plays an important role in natural product syntheses. Enyne metathesis, dienyne metathesis, cross enyne metathesis, and ROM of cycloalkene-yne have also been developed. Furthermore, skeletal reorganization using the transition metals or metallotropic rearrangement is a unique reaction. Novel procedures for the synthesis of the natural products and related compound, various complex molecules and macrocyclic compounds would be further developed using these various enyne metatheses. The retrosynthetic analysis of the natural products using these enyne metatheses would be completely different from that of the previous synthesis and the steps for the synthesis of these compounds would be shortened.
